# Peptide Hormone‐Mediated Regulation of Plant Development and Environmental Adaptability

**DOI:** 10.1002/advs.202506590

**Published:** 2025-07-10

**Authors:** Xin Li, Liping Zhu, Huiqin Wang, Xin Zhou, Manhong Wang, Libei Li, Feng Liu, Jie Sun, Guanghui Xiao

**Affiliations:** ^1^ National Key Laboratory of Cotton Bio‐breeding and Integrated Utilization School of Life Science Henan University Kaifeng Henan 475004 China; ^2^ Applied Research Institute of Life Sciences Xi'an International University Xi'an Shaanxi 710119 China; ^3^ College of Life Sciences Shaanxi Normal University Xi'an 710119 China; ^4^ Department of Poultry Science Mississippi State University Starkville MS 39762 USA; ^5^ College of Advanced Agriculture Sciences Zhejiang A & F University Lin'an 311300 China; ^6^ Key Laboratory of Oasis Eco‐agriculture College of Agriculture Shihezi University Shihezi Xinjiang 832000 China

**Keywords:** classification, environmental adaptability, immune response, plant development, plant peptides, plant reproduction

## Abstract

Peptide hormones are essential signaling molecules in plants, playing critical roles in regulating growth and development, stress responses, and crop genetic improvement. However, the full extent of their functions, functional redundancy among different peptide hormones, signaling mechanisms between ligand and receptor pathways, and peptide hormones‐mediated regulation of plant development and environmental adaptability are still not fully understood. In this study, recent advances in understanding the formation and functions of plant peptide hormones, distinguishing between non‐secretory and secretory peptides and their respective physiological roles are reviewed. These peptide hormones interact intricately with receptor kinases to regulate multiple signal transduction pathways. Plant peptide hormones are involved in cell proliferation and differentiation within both shoot apical and root apical meristems, as well as in the development of various organs. They also play a central role in plant reproduction, particularly in coordinating male‐female gametophyte interactions for successful fertilization. Furthermore, plant peptide hormones contribute to abiotic stress responses and immune responses against biotic stresses. An overview of the multifaceted roles of plant peptide hormones in growth, development, and environmental adaptability, emphasizing their importance in plant biology are presented. Understanding the complex functions of these peptide hormones lays the foundation for developing strategies to enhance crop resilience and productivity.

## Graphical abstract

In this review, recent advances in understanding the roles of plant peptide hormones in regulating growth, reproduction, and environmental adaptability are systematically summarized. It further elucidates the challenges in studying these peptides, such as their low abundance, functional redundancy, and complex signaling mechanisms, and highlights their potential applications in crop improvement. By leveraging peptide‐receptor interactions and post‐translational modifications, plants achieve precise regulation of physiological processes, offering innovative strategies for enhancing resilience and productivity in agriculture. The integration of peptide signaling networks with traditional hormone pathways provides a foundation for developing climate‐smart crops and sustainable agricultural practices. (Image elements are soured from http://BioRender.com.)

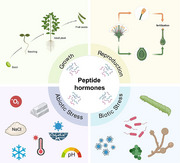

## 1 Introduction

Peptide hormones, prevalent in eukaryotic organisms, typically range 2–100 amino acids in length, that function as signaling molecules.^[^ ^1^ ^]^ Despite their small size, they play critical roles in regulating growth, development, stress responses, and cell‐to‐cell communication via receptor‐mediated signaling pathways.^[^ ^2^ ^]^ Since the first plant peptide hormone (systemin) was discovered, others have been reported to play significant roles in many aspects of plant life.^[^ ^3^ ^]^ Advances in peptidomics have led 1860 non‐conventional peptides (NCPs) being identified in the model dicot *Arabidopsis thaliana*, and 1993 members in the monocot *Zea mays*,^[^ ^4^ ^]^ suggesting that a substantial portion of the plant genome can be translated into bioactive molecules, and underscores the importance of these peptide hormones in plant systems various physiological as well as developmental processes.

The biosynthesis of plant peptide hormones includes ribosomal and non‐ribosomal synthesis, as well as protein processing.^[^ ^6^ ^]^ Thermostable cryptic peptides (TCPs) are released through proteolytic processes. Many plant peptide hormones are evolutionarily conserved across species and perform analogous functions.^[^ ^7^, ^8^ ^]^ CLAVATA3/ESR‐RELATED (CLE) peptide hormones occur in both monocots and dicots and are important for meristem maintenance and cell differentiation.^[^ ^9^ ^]^ Additionally, the receptors and downstream signaling components of plant peptide hormones are conserved across species. Receptor kinase FERONIA (FER), which interacts with Rapid Alkalinization Factors (RALF) peptide hormones to regulate plant reproduction and stress responses, occurs in multiple plant species.^[^ ^10^ ^]^

Most peptide hormones are encoded by precursor genes and are processed through proteolytic cleavage and post‐translational modifications to become mature, functional peptide hormones.^[^ ^11^ ^]^ Non‐secretory peptide hormones mainly function within the cell, whereas secretory peptide hormones are released into extracellular spaces via cellular secretion pathways. Non‐secretory peptide hormones are both extra‐ and intracellular, distinguished by the absence of a classical secretion signal peptide sequence. Intracellular non‐secretory peptide hormones function within the cell and do not follow traditional secretory pathways. Extracellular non‐secretory peptides, while acting outside the cell, are not secreted via classical pathways, but are released into the extracellular space through unconventional protein secretion (UPS). This release can occur either by directly crossing the plasma membrane (Type I) or via vesicular transport systems (Type III).^[^ ^12^ ^]^ In the extracellular space, these peptide hormones may undergo hydrolysis or additional post‐translational modifications to achieve their mature forms. Plant peptide hormones have many structures, including linear, cyclic, and peptide hormones with post‐translational modifications (PTMs) such as phosphorylation, glycosylation, and hydroxylation.^[^ ^1^, ^13^ ^]^ These peptide hormones are functionally diverse and regulate multiple processes such as cell division, elongation, differentiation, and overall plant morphogenesis.

Because of their low molecular weight and abundance, peptide hormones pose significant challenges to traditional separation and purification methods, leading to high difficulty, time‐consuming, and labor‐intensive research. Additionally, the sources of peptide hormones and their precursors are diverse and highly complex, with functional redundancy, which limits their discovery and physiological function through forward genetic studies of mutant phenotypes. Signal transduction mechanisms between upstream and downstream ligand‐receptor signaling pathways have yet to be fully deciphered. Reviews on peptide hormones help to systematically organize and summarize the latest research progress on them, providing a scientific basis for understanding their mechanisms of action in plant growth, development, and stress responses. We comprehensively review peptide hormones associated with plant development and stress responses. By examining how these peptide hormones exert their functions and interact with a plant's peptide and signaling networks, an improved understanding of plant communication is obtained.

Plant peptide hormones, as a novel class of signaling molecules, have garnered increasing attention for their pivotal roles in regulating plant growth and environmental adaptation. Unlike classical hormones, these peptide hormones function through specific binding to receptors such as PEPRs and PSY1R, thereby activating intercellular signaling pathways that govern growth, immunity, and stress responses.^[^ ^14^ ^]^ Recent studies have demonstrated their involvement in diverse processes, including pathogen defense, cell expansion, root differentiation, and nutrient transport.^[^ ^15^ ^]^ Moreover, peptide signals often intersect with traditional phytohormones like jasmonic acid and abscisic acid, acting as crucial mediators in balancing growth and defense, and representing valuable targets for improving crop resilience and breeding efficiency.^[^ ^16^ ^]^ In agricultural applications, their potential as eco‐friendly immune inducers and stress regulators.^[^ ^17^ ^]^ To this end, systematic studies on well‐characterized peptides with proven functions are essential for advancing our knowledge of plant adaptation mechanisms and driving innovation in precision breeding and green agriculture. Our study focuses on peptide hormones that have been experimentally validated and shown to play crucial roles in plant growth and development, and systematically summarizes recent advances in their functions in development, reproduction, and responses to biotic and abiotic stress. This study promotes interdisciplinary communication, advances the research and application of plant small peptides, and provides new ideas and strategies for crop improvement and agricultural production as well as develops strategies to enhance plant resilience to environmental challenges. This study promotes interdisciplinary communication, advances the research and application of plant peptide hormones, and provides new ideas and strategies for crop improvement and agricultural production as well as develops strategies to enhance plant resilience to environmental challenges.

We also provide a summary table to systematically summarize the detailed information of peptide hormones involved in this study, including the peptide names, length, receptor, site of action, mechanisms of action, functions, regulated developmental stage, species, and corresponding references (Table S1, Supporting Information).

## 2 Formation of Peptide Hormones in Plants

**Figure 1:**
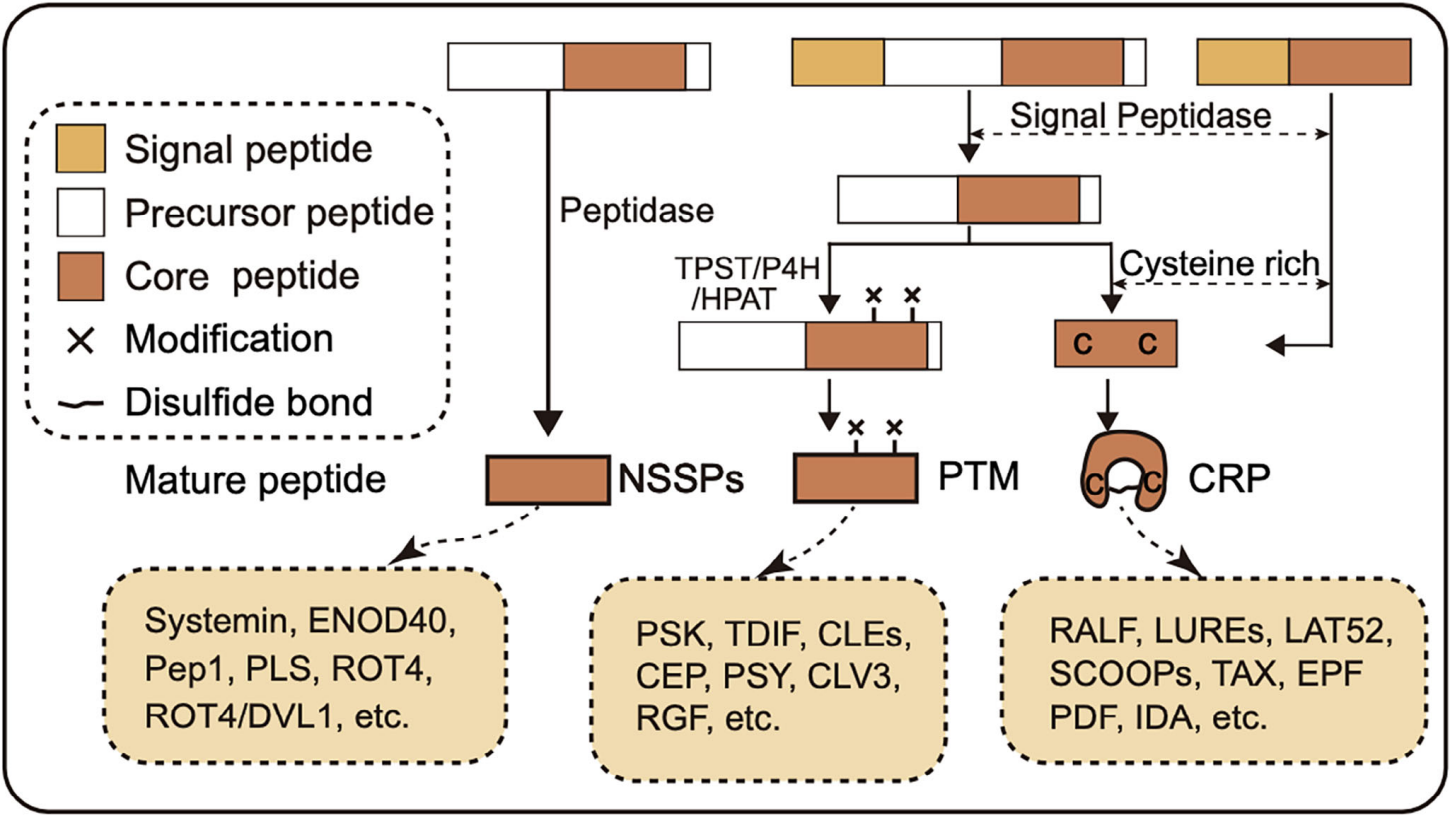
Production of Plant Peptides. Plant peptides include the non‐secretory small peptides (NSSPs), cysteine‐rich peptides (CRPs), and post‐translationally modified peptides (PTMs). NSSPs are generated by direct cleavage of their precursors through peptidases, resulting in mature peptides. CRPs are characterized by multiple cysteine residues that form disulfide bonds, which are crucial for maintaining the structural stability and functionality of the peptides. PTMs are produced through enzymatic processing and modification of precursor peptides. After initial synthesis, PTMs undergo additional enzymatic modifications to acquire biological activity. These modifications affect their interactions with receptors and other target proteins, influencing their overall function.

Plant peptide hormones can be categorized into non‐secretory and secretory peptides.^18^ Secretory peptide hormones encompass cysteine‐rich peptides (CRP) and PTMs. These peptide hormones are synthesized as precursor peptides that contain a signal peptide, which directs them to the target cell. Once there, they undergo enzymatic processing and modification to become mature, functional peptide hormones.^[^ ^20^ ^]^ CRPs are characterized by multiple cysteine residues that form disulfide bonds, essential for their stability and structural integrity. Conversely, PTMs require additional enzymatic modifications after synthesis to acquire biological activity and influence receptor interactions (**Figure** **1**).

PTM peptide hormones alter the physicochemical properties of peptides, affecting their net charge, hydrophilicity, and conformation to regulate the peptide's specific binding capacities to target proteins.^[^ ^21^ ^]^ Three key types of PTMs for peptide hormones have been identified: tyrosine sulfation, proline hydroxylation, and hydroxyproline arabinosylation.^[^ ^1^ ^]^ Tyrosine sulfation is catalyzed by tyrosylprotein sulfotransferase (TPST), which transfers a sulfate group from 3′‐Phosphoadenosine 5′‐Phosphosulfate (PAPS) to the phenolic group of tyrosine. Peptide signals with tyrosine sulfation include pentapeptide phytosulfokine (PSK), Plant peptide containing sulfated tyrosine (PSY), and Tracheary Element Differentiation Inhibitory Factor (TDIF) (Figure 1). Proline hydroxylation is mediated by Prolyl‐4‐Hydroxylase (P4H), a transmembrane protein found in the endoplasmic reticulum and Golgi apparatus. Excepting PSK, which lacks proline residues, other PTM peptide hormones contain hydroxyproline residues that are present in various secretory peptides such as PSY1, CLV3 (CLAVATA3), CLE2, CLE9, and CLE‐RS2, and are often further modified by O‐linked arabinose chains.^[^ ^22^, ^23^, ^24^ ^]^ Hydroxyproline arabinosylation involves the addition of arabinose to hydroxyproline residues, occurs primarily in a large family of extracellular proteins, and is catalyzed by Hydroxyproline O‐arabinosyltransferase (HPAT) that facilitates formation of a β‐link between the fourth position of hydroxyproline and arabinose.^[^ ^25^ ^]^ However, the detection of PTMs in small peptide hormones – especially sulfation and hydroxylation – remains challenging for mass spectrometric analysis due to the chemical instability of these modifications, technical limitations of MS, and database‐related interference.

CRP peptide hormones are a class of bioactive small peptides characterized by their multiple cysteine residues. Typically, CRPs possess a conserved signal peptide at the N‐terminus and an even number of cysteine residues at the C‐terminus.^[^ ^26^ ^]^ These cysteine residues can form intramolecular disulfide bonds, which are important for the peptide's 3D structure, stability, and function (Figure 1). Notable examples of cysteine‐rich peptide hormones include RALFs, defensins (PDF), LUREs, Taximin (TAX), Epidermal Patterning Factor (EPF), and Inflorescence Deficient in Abscission (IDA).^[^ ^27^, ^28^, ^29^, ^30^ ^]^ These peptide hormones play significant roles in regulating various physiological processes in plants, including reproduction, growth and development, and immune responses.

Compared with secretory peptides, non‐secretory peptide hormones are less‐extensively studied, but they regulate cellular functions and influence plant growth and development through intracellular signal transduction. Additionally, these peptide hormones can be released from damaged cells to directly contribute to plant defense and immune responses. Systemin and AtPEP1 serve as intercellular signals in plant defense mechanisms.^[^ ^31^, ^32^ ^]^ Early nodulin 40, POLARIS (PLS), and ROTUNDIFOLIA4 (ROT4)/DEVIL1 (ROT4/DVL1), are involved in multiple processes including plant nitrogen fixation, cell proliferation, and organ development.^[^ ^33^, ^34^, ^35^ ^]^ ROT4 enhances its interaction with the brassinosteroid signaling kinase Brassinosteroid Insensitive Signaling Kinase 5 (BSK5) through S‐acylation, which disrupts the signal transduction of the secretory peptide hormone Plant Elicitor Peptide 1 (PEP1), highlighting ROT4's significant role in regulating plant immunity.^[^ ^36^ ^]^ These findings suggest that non‐secretory peptides can modulate the activity of secretory peptide hormones, thus maintaining a dynamic balance in physiological processes. Together, non‐secretory and secretory peptide hormones coordinate to support plant growth, development, and responses to stress.

## 3 Plant Growth and Development Regulated by Plant Peptide Hormones

### 3.1 Shoot Apical Meristem

**Figure 2:**
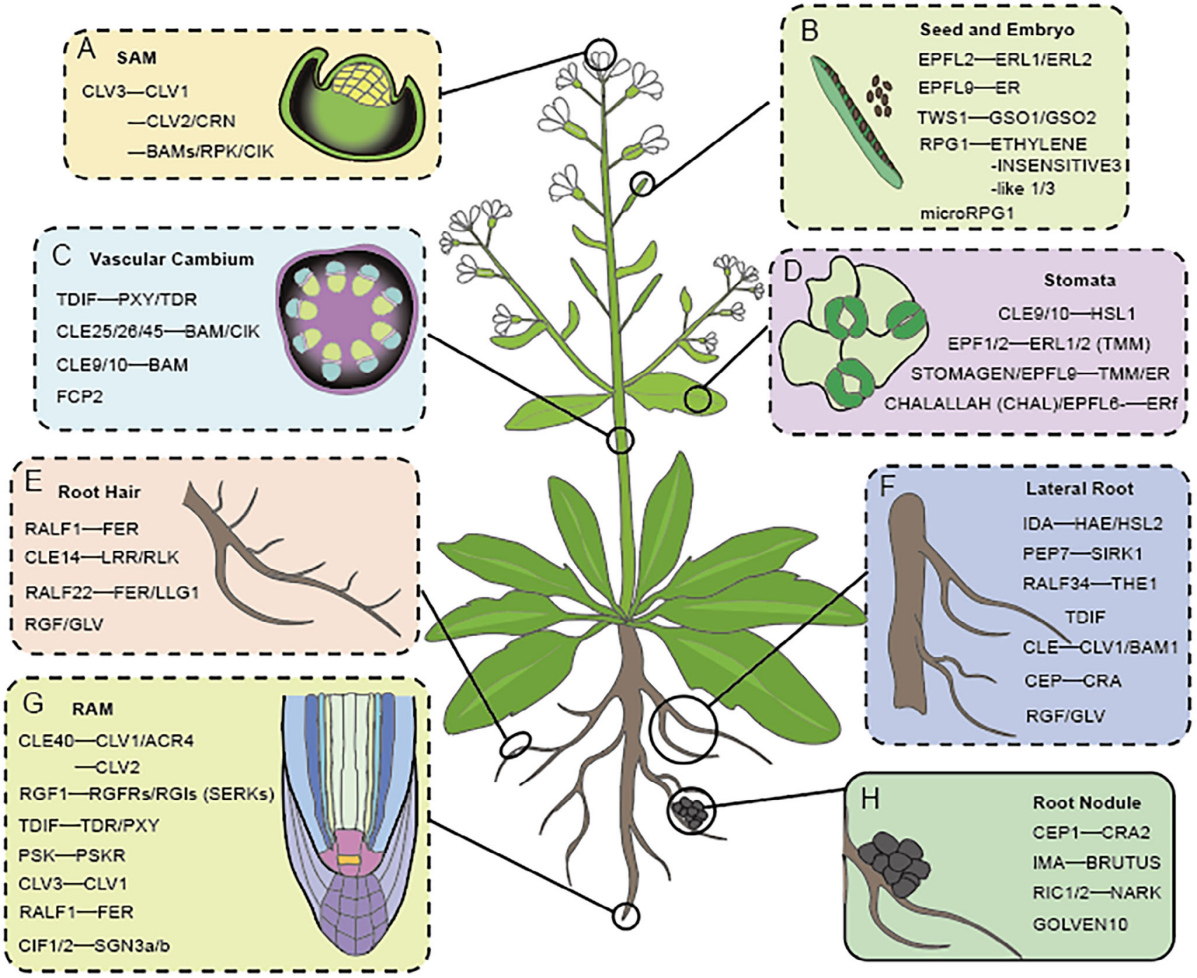
Distinct Function of Peptides in Various Plant Tissues. A) CLV3 interacts with different receptor kinases to regulate the maintenance and differentiation of the shoot apical meristem (SAM). B) EPFL2/9, TWS1, RPG1, and microRPG1 are involved in the developmental processes of plant seeds and embryos. C) CLE peptides, TDIF, and FCP2 play crucial role in the development of the vascular cambium. D) CLE9/10, EPF1/2, STOMAGEN/EPFL9, and CHALALLAH (CHAL)/EPFL6 are involved in signal transduction during stomatal development by binding to specific receptors. E) RALF1/22, CLE14, and RGF/GLV are involved in the developmental processes of the root hair. F) IDA, PEP7, RALF34, TDIF, CLE, CEP, and RGF/GLV play crucial role in the development of the lateral root. G) Various small peptide signals are essential for the root apical meristem (RAM). H) CEP1, IMA, RIC1/2, and GOLVEN10 are involved in the developmental processes of plant seeds and embryos.

The aerial part of a plant is primarily formed through the differentiation of the shoot apical meristem (SAM). Maintenance and differentiation of stem cells in the SAM are precisely regulated by the classic CLAVATA (CLV)–WUSCHEL (WUS) negative feedback loop, in which the *CLV3* gene, a member of the *CLAVATA3/EMBRYO SURROUNDING REGION‐RELATED* (*CLE*) gene family, is induced by the WUS transcription factor, which is specifically expressed in the central zone of the SAM (**Figure** **2**A). *CLV3* is expressed in these central zone stem cells, and secreted into the extracellular space. Once released, CLV3 diffuses through the tissue and binds to several membrane‐bound receptor‐like proteins (CLV1, CLV2, CORYNE (CRN), Barely Any Meristem (BAMs), RPK, and Clavata3 Insensitive Receptor Kinases (CIKs)), inhibiting expression of the *WUS* gene, thereby maintaining a stable SAM development.^[^ ^37^, ^38^, ^39^, ^40^, ^41^ ^]^

Orthologs of CLV3 have been identified in other species. In rice, the *FON2* gene, an ortholog of *Arabidopsis CLV3*, plays a role in negatively regulating stem cell proliferation.^[^ ^42^ ^]^ In tomato, the CLV3 homolog SlCLV3 is involved in apical meristem stem cell proliferation. When *CLV3* is mutated, the expression of the *CLE9* gene from the same family is upregulated to compensate for the loss of *CLV3* function, ensuring the robustness of stem cell development in the tomato apical meristem.^[^ ^43^, ^44^ ^]^ These findings underscore the importance of *CLV3* and its homologs in maintaining plant stem cell populations. These studies further demonstrate the functional conservation of the CLE‐CLV pathway in regulating meristem activity across diverse plant species.

### 3.2 Seed and Embryo

Plant peptide hormones are important for regulating various developmental processes in seeds and embryos of angiosperms. In *Arabidopsis*, members of the EPF/EPFL family, including EPFL2 and EPFL9, along with their receptor‐like kinases ERL1/ERL2 and ERECTA, coordinate ovule arrangement and fruit growth (Figure 2B). EPFL2 plays a pivotal role in regulating ovule spacing and fruit development, whereas EPFL9 primarily promotes fruit growth and antagonizes the function of EPFL2.^[^ ^45^ ^]^ Additionally, the *Arabidopsis* CIF family member TWISTED SEED 1 (TWS1) peptide hormone, in conjunction with GASSHO‐like receptor kinases GSO1 and GSO2, regulates the integrity of the embryonic cuticle.^[^ ^46^ ^]^ Kernel dehydration rate (KDR) is a crucial production trait that affects mechanized harvesting and kernel quality in maize. Quantitative trait locus (QTL), qKDR1, as a non‐coding sequence that regulates the expression of *qKDR1 REGULATED PEPTIDE GENE* (*RPG*), encodes a 31 amino acid micropeptide, microRPG1, which controls KDR by precisely modulating the expression of two genes, *ZmETHYLENE‐INSENSITIVE3‐like 1* and *3*, in the ethylene signaling pathway in the kernels after filling.^[^ ^47^ ^]^ Genome editing of these peptide hormones holds promise for improving crop seed and embryo development in breeding programs.

### 3.3 Vascular Cambium

The vascular cambium is important for initiating the development of the plant vascular system, and exhibits stem cell‐like properties.^[^ ^48^ ^]^ The CLE peptide hormone family plays an important role in vascular bundle development.^[^ ^49^ ^]^ TDIF (TADEDF/TDIF) peptide hormones, encoded by the *CLE41* and *CLE44* genes, are synthesized in the phloem of the plant stem and transported to the cambium and interact with their receptors, TDIF Receptor (TDR) and Phloem Intercalated With Xylem (PXY), to regulate the expression of downstream *WUSCHEL‐RELATED HOMEOBOX* (*WOX*) genes, specifically *WOX4* and *WOX14*.^[^ ^50^ ^]^ This inhibits differentiation of cambium stem cells to promote their proliferation, thereby maintaining the stem cell pool within the cambium (Figure 2C).

Although the specific receptors for the CLE41/CLE44 homologs have not been identified in rice, exogenous application or overexpression of these peptide hormones impacts late‐maturing xylem formation.^[^ ^51^ ^]^ During root vascular development, phloem‐enriched Dof transcription factors, known as phloem‐Dof, activate CLE25/26/45 peptide hormones, which are then secreted into the extracellular space and captured by BAM/CIK receptors to inhibit the stability of Dof proteins, thus preventing procambium differentiation and phloem formation.^[^ ^52^ ^]^ Additionally, CLE9/10 peptide hormones, by binding to BAM‐like receptor kinases, inhibit the circumferential division of xylem precursor cells, thereby regulating root xylem development.^[^ ^53^ ^]^ The CLE peptide family (including TDIF, CLE25/26/45, and CLE9/10) plays a central regulatory role in vascular cambium stem cell maintenance and xylem/phloem differentiation through receptor kinases (such as TDR/PXY and BAM/CIK) and downstream WOX gene networks. Although some receptors remain unidentified in crops like rice, their functional conservation suggests potential for cross‐species applications.

### 3.4 Stomata

Stomata are important for gas exchange between plants and their environment, and peptide hormones play a significant role in their development through receptor‐mediated signaling. The CHALALLAH (CHAL) peptide hormone, also known as EPFL6, interacts with the ERECTA (ER) receptor to inhibit stomatal formation. Too Many Mouths (TMM) can counteract the inhibitory effects of CHAL on epidermal patterning^[^ ^54^ ^]^ (Figure 2D). EPF1 and EPF2 peptide hormones bind to the ER family receptor kinases ERL1 and ERL2, and the co‐receptor TMM, to inhibit stomatal development and reduce stomatal density.^[^ ^55^ ^]^ In rice, overexpression of EPF homologs *OsEPF1* and *OsEPF2* significantly decreases stomatal density.^[^ ^56^ ^]^ Conversely, the EPFL family peptide hormone Stomagen (also called EPF‐LIKE9) promotes stomatal development by competitively binding to the TMM/ER receptor and reducing the phosphorylation of downstream components caused by EPF2.^[^ ^55^ ^]^ In *Arabidopsis*, CLE9 and CLE10 peptide hormones bind to the HAESA‐LIKE 1 (HSL1) receptor to regulate the division of stomatal lineage cells.^[^ ^53^ ^]^ Plant stomatal development is finely tuned by small peptide hormones (CHAL/EPFL6, EPF1/2, STOMAGEN, CLE9/10) and their receptors (ER family, TMM, HSL1) forming a regulatory network. Targeted modification of these peptide‐receptor systems could allow precise control of stomatal density to enhance drought tolerance (via reduced water loss) or photosynthesis (via increased CO₂ uptake), offering innovative approaches for climate‐smart crop breeding.

### 3.5 Root Hair

In *Arabidopsis*, RALF1 forms a complex with its receptor, the FER kinase, modulating protein synthesis by promoting the phosphorylation of eIF4E1 (eukaryotic Initiation Factor 4E1). This phosphorylation enhances eIF4E1's affinity for mRNAs, including those of Rho GTPases in plants 2 (ROP2) and RHD‐6‐LIKE 4 (RSL4), which are important for root hair cell polarity and growth (Figure 2E). The polar expression of the *RALF1*‐*FER* complex in root hairs facilitates the polar localization of eIF4E1, potentially controlling the localized translation of ROP2. High levels of RSL4 provide negative feedback on *RALF1* expression by directly binding to the *RALF1* promoter, ultimately determining root hair size.^[^ ^57^, ^58^ ^]^ CLE14 induces root hair formation in *Arabidopsis*.^[^ ^59^ ^]^ Treatment with synthetic CLE14 peptide hormone promotes root hair growth and reduces expression of the non‐hair cell fate determination gene *GLABRA2* (*GL2*) in *Arabidopsis* roots. CLE14 also suppresses expression of *GL2* in rice and tomato, indicating a conserved function of CLE14 across species.^[^ ^60^ ^]^

RALF22 plays a structural and signaling role in root hair cell wall assembly. Together with Leucine‐Rich Repeat Extensin 1 (LRX1), it directs the compaction of charged pectin polymers at the root hair tip into periodic circumferential rings. Free RALF22 induces the formation of a complex with LORELEI‐LIKE‐GPI‐ANCHORED Protein 1 (LLG1) and FER, triggering adaptive cellular responses.^[^ ^61^ ^]^ The peach RGF/GLV signaling peptide pCTG134 is involved in a regulatory circuit that sustains auxin and ethylene actions, influencing root hair growth and lateral root development.^[^ ^62^ ^]^

### 3.6 Lateral Root

The peptide hormone IDA, which is induced during the early stages of lateral root primordium formation, is recognized by the receptors HAESA (HAE) and HAESA‐Like 2 (HSL2). Activation of the Mitogen‐activated protein kinase3/6 signaling cascade triggers expression of genes associated with cell wall remodeling, facilitating cell wall degradation, and thereby promoting lateral root development.^[^ ^63^ ^]^ Pollen‐expressed peptide 7 (PEP7), as a peptide hormone ligand for the receptor kinase Sucrose‐Induced Receptor Kinase 1 (SIRK1), modulates water influx mediated by plant aquaporins. The PEP7/SIRK1/QSK1 (Quantum Sick 1) complex regulates lateral root growth^[^ ^64^ ^]^ (Figure 2F).

The Changchun flower Receptor‐Like Kinase 1‐Like (CrRLK1L) family receptor THEEUS1 (THE1) recognizes the RALF34 peptide hormone to regulate lateral root development.^[^ ^65^ ^]^ TDIF peptide hormones influence root growth by affecting auxin homeostasis and *PIN‐FORMED* (*PIN*) expression in *Arabidopsis*.^[^ ^66^ ^]^ In poplar trees, TDIF regulates auxin accumulation and modulates auxin sensitivity to enhance both adventitious and lateral root formation.^[^ ^67^ ^]^

In *Arabidopsis*, the CLE1‐CLE7 peptide hormone family and their receptor CLV1 are involved in the negative regulation of lateral root development under nitrate limitation. CLE3 and its homologs interact with the CLV1/BAM1 receptor complex, modulating expression of *Lateral Organ Boundaries Domain/Asymmetric Leaves2‐Like (LBD/ASL)* genes to control lateral root formation. Mutants with a single alteration in *CLE3* have elongated lateral roots, whereas plants overexpressing both *CLE2* and *CLE3* have reduced lateral root length.^[^ ^68^ ^]^ CEP‐CRA acts as a negative regulator of root development, slowing primary root growth and reducing lateral root formation.^[^ ^69^ ^]^

### 3.7 Root Apical Meristem

The underground part of plants primarily develops from the root apical meristem (RAM), wherein the CLV3 peptide hormone downregulates expression of the homeodomain transcription factor *WUS* by binding to the receptor CLV1, which forms a negative feedback loop that regulates stem cell proliferation.^[^ ^70^ ^]^ CLE40, a close homolog of CLV3, regulates expression of the WUS homolog gene *WUSCHEL‐RELATED HOMEOBOX 5* (*WOX5*) in the quiescent center by binding to the receptor kinase Arabidopsis Crinkly 4 (ACR4)/CLV1, thus promoting stem cell differentiation.^[^ ^71^, ^72^ ^]^ CLE40 can also enhance *WOX5* expression in the proximal meristem through the CLV2 receptor, determining the quiescent center's position in the root.^[^ ^73^ ^]^

In rice, FON2‐like CLE Protein 2 (FCP2), functionally homologous to CLE41/CLE44, regulates the *QUIESCENT‐CENTER‐SPECIFIC HOMEOBOX* (*QHB*) gene and is involved in RAM development.^[^ ^51^ ^]^ The RGF1 peptide hormone is recognized by the receptors Receptor of RGFs/RGF1 Insensitives (RGFRs/RGIs) and the co‐receptors Somatic Embryogenesis Receptor Kinases (SERKs), promoting expression of the downstream *RGF1 Inducible Transcription Factor 1* (*RITF1*) gene, which leads to redistribution of reactive oxygen species (ROS) in the root development area and regulates the stability of key root development regulators (Figure 2G). The RGF1–RGFRs/RGIs–SERKs pathways are crucial for maintaining stem cell activity.^[^ ^74^, ^75^ ^]^

PSK functions by binding to the PSK Receptor (RPSK) and the co‐receptor Somatic Embryogenesis Receptor‐like Kinase (SERK), influencing stem cell maintenance and morphogenesis in the root meristem.^[^ ^76^ ^]^ Rapid Alkalinization Factor 1 (RALF1) reversibly inhibits primary root growth through apoplastic alkalinization, which results from net H^+^ influx induced by RALF1 and mediated by the Receptor FERONIA (FER).^[^ ^77^ ^]^

The casparian strip, located on the inner side of epidermal cells in the root tip and developed from root cap cells, controls substance movement into and out of cells.^[^ ^78^ ^]^ Casparian strip integrity factors 1/2 (CIF1/2) and the receptor‐like kinases SCHENGEN3a/b (SGN3a/b) are involved in regulating the formation of the casparian strip in both endodermal and non‐endodermal cells in rice.^[^ ^79^ ^]^ Although the roles of CLV3/CLE40‐WOX5 and RGF1‐RGFR pathways in root apical meristem (RAM) maintenance are well established, the crosstalk between different peptide signaling pathways (e.g., RALF1‐FER and PSK‐SERK interactions) and species‐specific mechanisms (e.g., differences between rice FCP2‐QHB and Arabidopsis CLE40‐WOX5) remain to be fully elucidated.

## 4 Root Nodule

Under low nitrogen conditions, the *Medicago truncatula* Compact Root Architecture 2 (MtCRA2) receptor kinase activated by MtCEP1 interacts with and phosphorylates the ethylene signaling component MtEIN2, preventing its cleavage (Figure 2H). This inhibition of the ethylene signaling pathway locally enhances the root's sensitivity to rhizobia, which are essential for nitrogen‐fixing nodule formation.^[^ ^57^ ^]^ Additionally, the CEP1‐CRA2 interaction upregulates the expression of the nitrate transporter *MtNRT2.1*, promoting root nodule formation.^[^ ^80^ ^]^ Expression of *CEP1* is regulated by transcription factor MtNLP1.^[^ ^81^ ^]^

The peptide hormone GOLVEN10 affects root development and nodule taxis in *M. truncatula*.^[^ ^82^ ^]^ In *Lotus japonicus*, IRON MAN (IMA) peptide hormones regulate root nodulation and nitrogen homeostasis by supplying iron according to internal nitrogen status. IMA1 and IMA2 peptide hormones function through the receptor kinase BRUTUS (BTS).^[^ ^83^, ^84^ ^]^

In soybean, GmRIC1/2, classified as *Rhizobium*‐induced CLE peptide hormones, are produced in response to rhizobium signals and are involved in the development of nodule primordia.^[^ ^85^ ^]^ GmRIC1/2 are translocated to above‐ground tissues, where they interact with and activate the receptor kinase GmNARK. The GmRIC1/2–GmNARK complex plays a role in the long‐distance inhibition of additional nodule formation, a mechanism associated with the autoregulation of nodule number.^[^ ^86^ ^]^

Plants precisely regulate root architecture and symbiotic nitrogen fixation through peptide‐receptor kinase modules (e.g., MtCEP1‐MtCRA2, GmRIC1/2‐GmNARK, IMA‐BRUTUS), forming an integrated network of local responses and long‐distance feedback. Future research could target these pathways using gene editing or synthetic peptide delivery technologies to optimize root‐microbe interactions in crops, enabling efficient nitrogen fixation under low‐nitrogen conditions and providing innovative strategies for sustainable agriculture.

## 5 Role of Peptides in Plant Reproduction

**Figure 3:**
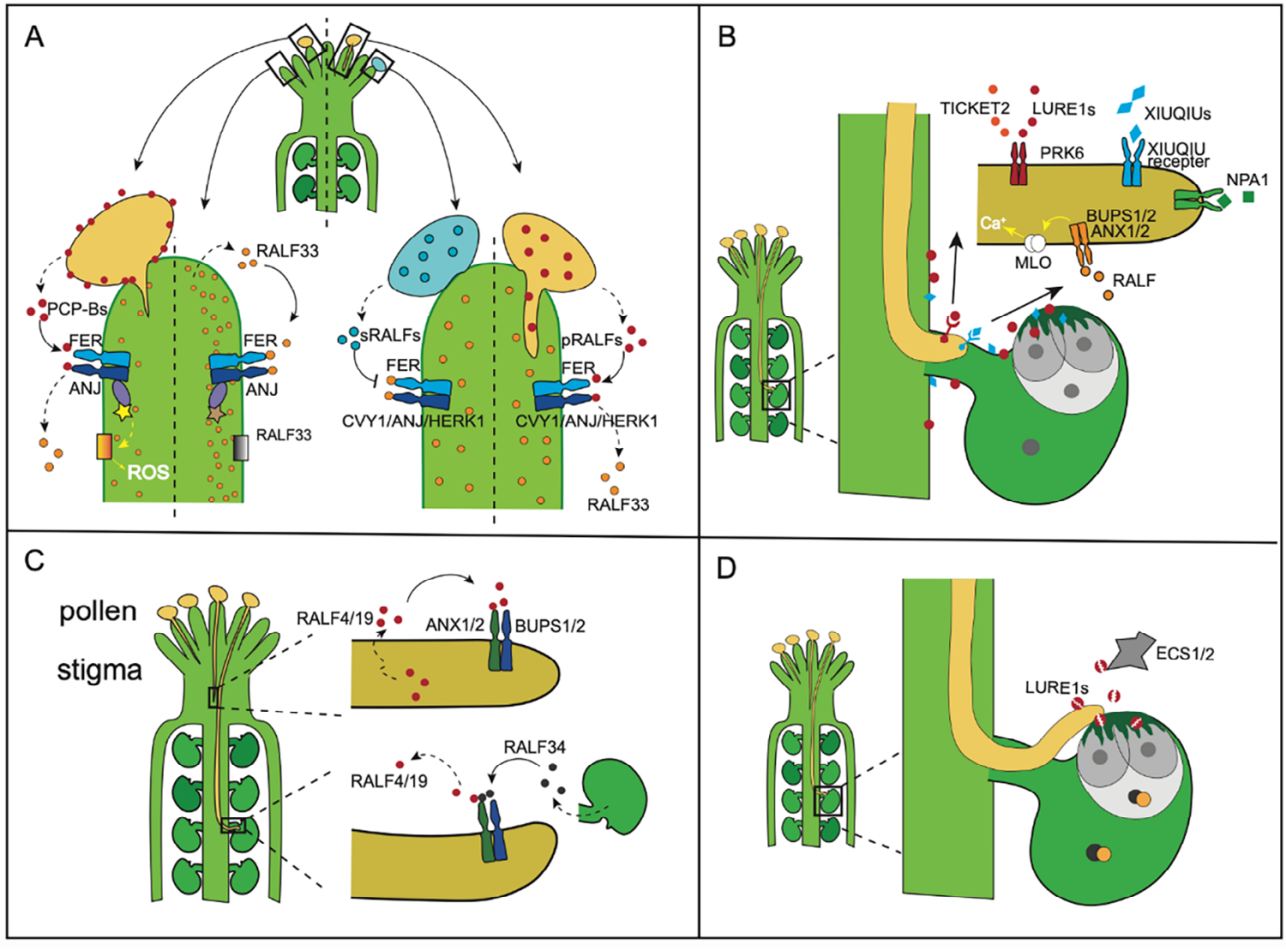
Plant Peptide Mediated Pollen‐Stigma Recognition and Germination. A) Pollen coat protein‐B class peptides (PCP‐Bs) from the pollen coat competitively bind to receptors on the stigma alongside self‐secreted RALF33 peptides to modulate pollen hydration and germination. Compatible pollen‐derived peptides (pRALFs) disrupt the lock established by self‐RALFs (sRALFs) and their receptors, thereby facilitating the penetration of the pollen tube into the stigma. B) Polar growth of the pollen tube: the pollen tube grows downward through the style toward the ovule after germination. Various small peptides released by the ovule, including TICKET2, LURE1s, XIUQIUs, and RALF peptides, interact with the tip of the pollen tube to guide the pollen tube's growth direction, ensuring it reaches the ovule. C) Pollen tube rupture: Upon arrival at the ovule, the female‐secreted peptide RALF34 competitively replaces the pollen tube‐derived peptide RALF4/19 to bind to its receptor, which triggers the rupture of the pollen tube and the release of sperm cells, preparing for double fertilization. D) Fusion of male and female gametes: Following fertilization, aspartyl endopeptidases ECS1 and ECS2 are secreted from the egg cell into the extracellular space. These endopeptidases specifically cleave the LURE1 peptide induced by the pollen tube, preventing re‐fertilization of the egg cell and ensuring successful completion of the fertilization process.

Plant peptide hormones play important roles in coordinating male–female gametophyte interactions for successful flowering plant reproduction through receptor‐like kinase‐mediated signaling.^[^ ^87^ ^]^ Several key events are involved in these process: pollen–stigma recognition and germination, polar growth of the pollen tube, pollen tube rupture and sperm cell release, and fusion of male and female gametes. Peptide hormones are essential messengers throughout these stages. Initially, the stigma must recognize the deposited pollen, allowing only compatible pollen to germinate and form a pollen tube. Research on *Arabidopsis* has shown that pollen uses Pollen Coat Protein‐B class peptides (PCP‐Bs) to compete with RALF23/33 for the ANJ‐FER complex on the stigma. These PCP‐Bs are able to interact with the ANJEA and FERONIA (ANJ‐FER) receptor kinase complex, inhibiting the reactive oxygen species (ROS) level maintained by the RALF33/ANJEA‐FER receptor kinase signaling pathway in the stigma, which further affects pollen hydration and germination (**Figure** **3**A). Furthermore, receptor‐like kinases such as FER/CURVY1/ANJEA/HERCULES Receptor Kinase 1 and cell wall proteins LRX3/4/5 on papilla cell surfaces interact with autocrine stigmatic RALF1/22/23/33 peptide ligands (sRALFs) to create a block that prevents the penetration of undesired pollen tubes. Compatible pollen‐derived RALF10/11/12/13/25/26/30 peptides (pRALFs) function as a key, outcompeting sRALFs and allowing the successful penetration of the pollen tube.^[^ ^88^ ^]^

After the stigma and pollen undergo mutual recognition, the pollen tube germinates on the stigma and extends downward, carrying two sperm cells through its channel. Concurrently, the ovule releases various peptide hormone signals, known as tip‐growing pollen tube (PT) attractants from synergid cells (SCs), which attract the pollen tube. Guided by these signals, the pollen tube alters its growth direction, penetrating the style, and moves toward the ovule.^[^ ^89^ ^]^ Another peptide hormone, LURE, which is cysteine‐rich and secreted by synergid cells, induces the asymmetric distribution of Pollen Receptor‐Like Kinase 6 (PRK6) at the tip of the pollen tube. This signal is transduced via Rho of Plants Guanine Nucleotide Exchange Factor 12 (ROPGEF12) and the cytosolic kinases Lost In Pollen Tube Guidance 1 and 2 (LIP1/2), modulating the oriented growth of the pollen tube.^[^ ^90^, ^91^, ^92^ ^]^ LURE1 also enhances the competitive ability of conspecific pollen tubes, promotes genetic isolation from closely related species, and ensures directed growth of conspecific pollen.^[^ ^93^ ^]^ Additionally, PRK6 interacts directly with the peptide TICKET2, further mediating the attraction of pollen tubes (Figure 3B).

In *Arabidopsis*, the phosphatidylinositol‐anchored proteins Lorelei‐Like Gpi‐Anchored Proteins 2 and 3 (LLG2/3) serve as companion proteins for the ANXUR/BUPS receptor kinase, which assist in transporting these receptors from the cytoplasm to the pollen tube's apical cell membrane and act as co‐receptors in the ANXUR/BUPS complex, responding to Rapid Alkalinization Factor 4/19 (RALF4/19) to regulate pollen tube polarized growth (Figure 3B). In monocots, ZmRALF regulates cell wall integrity during maize pollen tube growth.^[^ ^94^ ^]^ Disruption of the RALF signaling pathway abolishes the cytosolic Ca^2+^ gradient in the pollen tube, indicating that Ca^2+^ signaling is downstream of the RALF signaling pathway. The Mildew Resistance Locus O (MLO) family proteins MLO1, 5, 9, and 15 act as Ca^2+^ channels necessary for Ca^2+^ influx and pollen tube integrity. RALF peptide hormones derived from pollen tubes bind to their receptors to establish the pollen tube Ca^2+^ gradient through MLO channel activation.^[^ ^95^ ^]^ To enhance the chances of successful fertilization, the XIUQIU peptide hormone can attract pollen from closely related species to grow toward the ovule (Figure 3B). In maize, the egg‐apparatus‐secreted polymorphic peptide hormone ZmEA1 attracts maize pollen tubes in vitro and arrests their growth at higher concentrations. When expressed in *Arabidopsis* synergid cells, ZmEA1 guides pollen tubes in vitro toward the micropylar opening of the ovule in a species‐preferential manner. This demonstrates how peptide hormones can regulate reproductive isolation disorders controlled by pollen tube‐guided organelles.^[^ ^96^ ^]^ Defensin‐like proteins of the CRP810 family, including LUREs, XIUQIUs, and TICKETs, are among the few identified pollen tube attractants in dicots.^[^ ^91^, ^93^, ^97^ ^]^ The peptide hormone Non‐Defensin Peptide Attractant 1 (NPA1) is distinct from these and represents a novel non‐defensin‐like peptide hormone, illustrating the diversity of attraction signals in biological systems.^[^ ^98^ ^]^

During the growth of the pollen tube toward the embryo sac, the BUPS–ANX receptor complex on the pollen tube membrane receives the peptide hormone signal RALF4/19, which is secreted by the pollen tube itself to maintain pollen tube cell integrity (Figure 3C). Upon reaching the embryo sac, the peptide hormone RALF34, secreted by the female side, competitively replaces RALF4/19 bound to the BUPS‐ANX receptor complex, which further triggers the rupture of the pollen tube and the release of sperm cells, preparing for double fertilization.^[^ ^99^ ^]^ After fertilization, aspartyl endopeptidases ECS1 and ECS2 in the egg cell are secreted from the cortical network at the egg cell's apical domain into the extracellular space (Figure 3D). These endopeptidases specifically cleave the LURE1 peptide hormone induced by the pollen tube, thereby preventing re‐fertilization of the egg cell.^[^ ^100^ ^]^

To enhance genetic diversity, plants have evolved a self‐incompatibility mechanism that operates during pollen tube guidance and identification prior to fertilization. The S‐locus Cysteine‐Rich Protein/S‐locus Protein 11 (SCRp/SP11), a CRP with eight cysteines, is a subclass of defensin‐like proteins specifically expressed in the tapetum of anthers and developing pollen. Before binding to the SCRp/SP11 peptide hormone, the kinase activity of S‐Receptor Kinase (SRK) is inhibited by thioredoxin‐h‐like (THL)‐1/2. Upon binding of the peptide hormone, SRK dissociates from THL1/2, leading to activation of the kinase, which further initiates a signaling cascade that inhibits pollen hydration and triggers a self‐incompatibility response.^[^ ^101^ ^]^ These studies reveal that plant peptide hormones play pivotal roles in reproductive processes, with their precise regulatory functions spanning pollen‐pistil interactions and fertilization control. Notably, the self‐incompatibility mechanism mediated by peptide hormones during pre‐fertilization pollen tube guidance and recognition provides a foundation for artificially manipulating peptide‐ligand pathways. This finding could potentially overcome self‐incompatibility barriers and facilitate the breeding of inbred crop lines.

## 6 Peptide Hormones Play Key Roles in Regulating Plant Adaptation to Environment

### 6.1 Oxidation Tolerance

**Figure 4:**
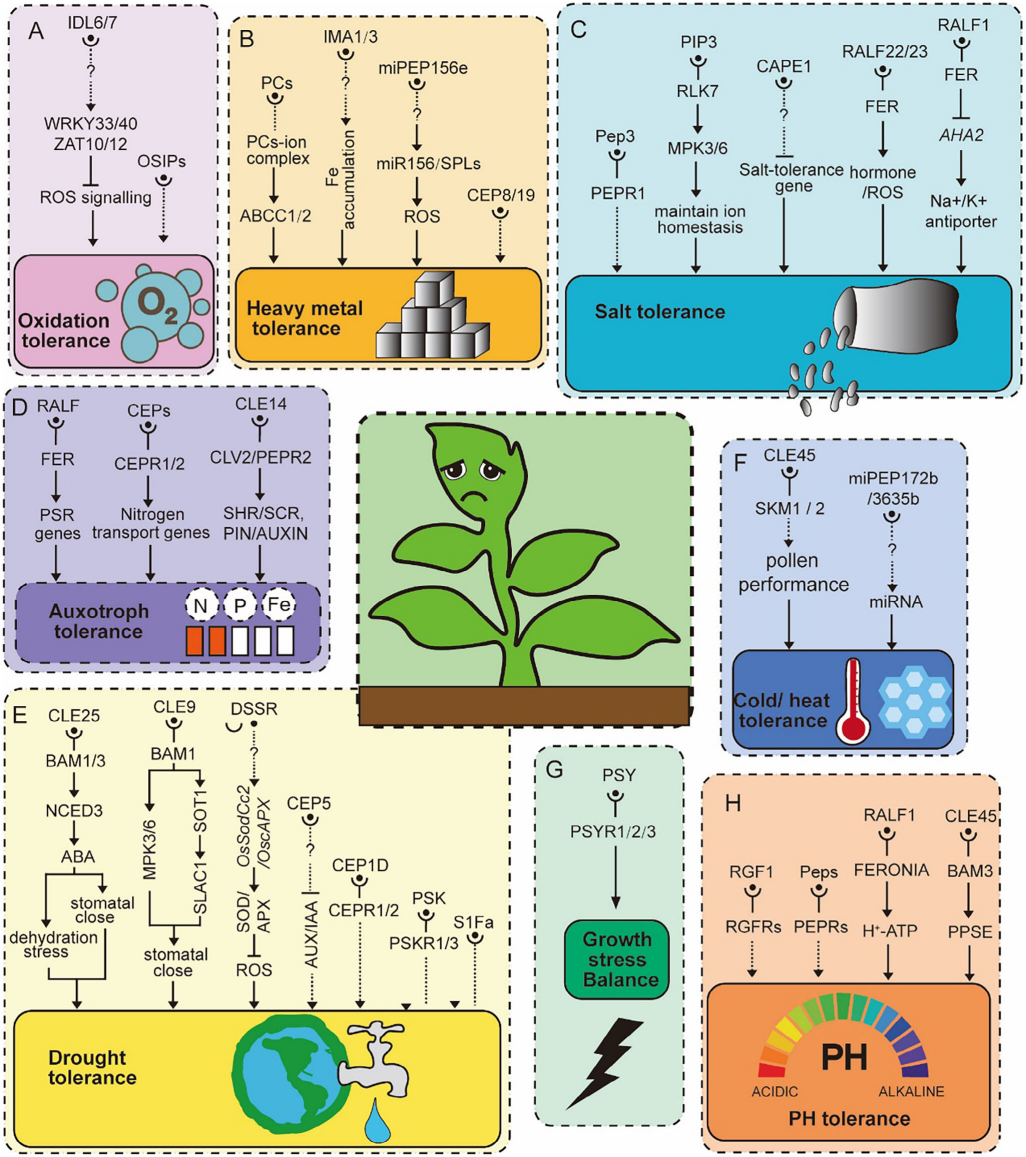
The Role of Peptides in Plant Resistance to Abiotic Stress. A) IDL6/7 and OSIPs involved in oxidation tolerance. B) PCs, IMA1/3, miPEP156e, and CEP8/19 regulate the heavy metal tolerance. C) Pep3, PIP3, CAPE1, RALF22/23, and RALF1 are involved in the salt tolerance. D) RALF, CEPs, and CLE14 play crucial role in the auxotroph tolerance. E) CLE, DSSR, CEP, PSK, and S1Fa are involved in the drought tolerance. F) CLE45 and miPEP172b/3635b regulate the temperature tolerance. G) PSY plays crucial role in the balance between stress tolerance and growth. H) RGF1, Peps, RALF1 and CLE45 regulate pH sencing and regulation. Solid lines denote direct regulation, whereas dashed lines represent indirect regulation involving multiple steps or unknown components.

Hydrogen peroxide (H₂O₂), a key ROS, can induce oxidative stress and cause damage in plants when present in excess. In *Arabidopsis*, three Oxidative Stress‐Induced Peptides (OSIPs) have been identified, and their overexpression in yeast significantly enhances the tolerance to H_2_O_2_.^[^ ^102^ ^]^ IDL7 and IDL6 act as negative regulators of stress‐induced ROS signaling in *Arabidopsis* (**Figure** **4**A). H_2_O_2_ rapidly induces the expression of *IDL7*, and *idl6*/*idl7* double mutant exhibit reduced cell death when exposed to extracellular ROS.^[^ ^103^ ^]^ These findings reveal the dual regulatory role of small peptide hormones in oxidative stress homeostasis, providing novel targets for developing stress‐resistant crops.

### 6.2 Heavy Metal Tolerance

Phytochelatins (PCs) are plant‐derived metal chelators that are complexed with heavy metal ions through the thiol groups of their cysteine residues, reducing the cytosolic concentration of free heavy metals and mitigating their cytotoxic effects.^[^ ^104^ ^]^ The PC‐metal complexes are sequestered within the vacuole by ABCC1 and ABCC2 transporters on the tonoplast, enhancing plant tolerance to cadmium and arsenic stress (Figure 4B). Ectopic expression of IMA peptide hormone genes imparts tolerance to cadmium stress by activating the iron deficiency response in *Arabidopsis*.^[^ ^105^ ^]^ Additionally, a regulatory small peptide (miPEP156e) encoded by pri‐miR156e modulates the expression of *miR156* and its target *SPL* genes, thereby enhancing miR156‐mediated cadmium tolerance in rice.^[^ ^19^ ^]^ Under cadmium stress, the *BrCEP8/19* gene is upregulated in *Brassica rapa*;^[^ ^106^ ^]^ however, the precise mechanisms behind this regulation require further investigation.

### 6.3 Salt Tolerance

Salt stress limits plant growth and productivity.^[^ ^107^, ^108^, ^109^ ^]^ Under saline conditions, the precursor gene *prePIP3*, responsible for synthesizing the Pamp‐Induced Secreted Peptide 3 (PIP3) peptide hormone, exhibits enhanced expression.^[^ ^110^ ^]^ The mature PIP3 peptide hormone is secreted into the extracellular space, where it forms a complex with Receptor‐Like Kinase 7 (RLK7) to activate the MPK3 and MPK6 signaling cascade, which modulates salt tolerance.^[^ ^110^ ^]^ Salt stress also upregulates expression of *AtPEP3* gene, whose product AtPep3 is recognized by PEP Receptor 1 (PEPR1) (Figure 4C). This interaction plays a role in salinity stress tolerance, with plants carrying a silenced *AtPep3* gene showing increased sensitivity to salt stress in *Arabidopsis*.^[^ ^111^ ^]^ Furthermore, levels of AtCAPE1 are elevated systemically under saline conditions, which negatively regulates salt‐stress tolerance by suppressing expression of genes involved in osmotic adjustment, detoxification, stomatal regulation, and membrane protection in *Arabidopsis*.^[^ ^112^ ^]^

The LRXs‐RALFs‐FER signaling module regulates plant growth and salt stress tolerance.^[^ ^108^ ^]^ Specifically, LRX3/4/5 proteins, essential for promoting plant growth and maintaining cell wall integrity, interact with RALF22/23 peptide hormones, blocking their interaction with the receptor kinase FER (Figure 4C). Under salt stress, LRX3/4/5 proteins dissociate from the RALF peptide hormones because of changes in the cell wall to allow RALF22/23 to bind to the FER receptor, which then modulates hormonal equilibrium and ROS accumulation.^[^ ^113^ ^]^ Additionally, RALF1‐FER signaling enhances salt toxicity by increasing Na^+^ accumulation and decreasing K^+^ accumulation.^[^ ^114^ ^]^ FER modulates the stability of the Shikimate Oxidoreductase Homolog 1 (SHM1) protein through phosphorylation to regulate the metabolic flux of the photorespiratory pathway under salt stress. Consequently, FER‐mediated regulation of SHM1 contributes to the plant's adaptation to high salinity conditions, facilitating plant survival and growth in saline environments.^[^ ^115^ ^]^ These studies revealed that plants employ multiple peptide hormone signaling pathways (e.g., PIP3‐RLK7, AtPep3‐PEPR1, and RALF‐FER modules) to coordinately respond to salt stress, regulating critical physiological processes including ion homeostasis, ROS balance, and cell wall integrity. These findings highlight the central role of peptide hormones in plant salt stress adaptation, although the interaction mechanisms between different pathways and their conservation in crops remain to be further elucidated.

### 6.4 Auxotroph Tolerance

Under phosphorus limitation, the CLE14 peptide hormone recognized by the CLV2 and PEPR2 receptors initiates cellular differentiation within the RAM to suppress the SHORTROOT (SHR)/SCARECROW (SCR) and PIN/AUXIN signaling pathways, resulting in the shortening of the primary root and enhanced proliferation of lateral and adventitious roots.^[^ ^116^ ^]^ This adjustment in root architecture helps plants better acclimate to low‐phosphorus environments. The transcription factor PHR1, associated with the phosphate starvation response (PSR), directly binds to and enhances transcription of *RALFs* under phosphate limitation.^[^ ^117^ ^]^ Peptide hormones produced by these *RALF* genes inhibit plant immune responses via the receptor kinase FER. This modulation of the root‐associated microbial community helps to alleviate the effects of phosphate scarcity.^[^ ^118^ ^]^ Under nitrogen deprivation, the expression of *C‐terminally Encoded Peptides* (*CEPs*) in roots is induced by nitrogen starvation signals, eliciting a nitrogen deficiency response in the aerial parts of the plant (Figure 4D). Upon translocation to the shoot, CEPs interact with their receptors CEP Receptor 1 (CEPR1) and CEPR2, leading to upregulation of genes associated with nitrogen transport to facilitate the signaling cascade that addresses nitrogen starvation.^[^ ^119^, ^120^ ^]^ Plants dynamically regulate root architecture (inhibiting primary root growth while promoting lateral root formation) and nitrogen/phosphorus transporter gene expression through peptide hormone signaling pathways (e.g., CLE14‐CLV2/PEPR2 and CEP‐CEPR1/2) under phosphorus/nitrogen deficiency, thereby achieving nutrient stress adaptation. These findings demonstrate the pivotal role of peptide‐mediated “root‐shoot communication” in plant nutrient sensing. However, the crosstalk mechanisms between different nutrient signaling pathways and their evolutionary conservation in crops remain to be fully elucidated.

### 6.5 Drought Tolerance

Intensifying global drought conditions significantly impact plant growth, and significantly reduce crop yield.^[^ ^121^, ^122^ ^]^ Many studies have reported the importance of plant peptide hormones in responses to drought stress. CLE family peptide hormone CLE25 detects water deficiency signals in roots and is transported over long distances via the vascular system to the leaves by binding to the receptor BAM1/3, triggering the biosynthesis of NCED3, a key enzyme in abscisic acid (ABA) production (Figure 4E). This process leads to stomatal closure and enhances plant drought tolerance.^[^ ^123^ ^]^ Similarly, CLE9, another member of the CLE family, binds to the BAM1 receptor to initiate a signaling cascade that activates ABA‐responsive elements in guard cells, such as OST1 and SLAC1, as well as the MAP kinases MPK3 and MPK6, culminating in stomatal closure in response to drought stress.^[^ ^124^, ^125^ ^]^

In rice, the signaling peptide Drought and Salt Stress Response‐1 (OsDSSR1) activates expression of genes associated with ROS scavenging, specifically *OsSodCc2* and *OscAPX*, to increase the enzymatic activity of Superoxide Dismutase (SOD) and Ascorbate Peroxidase (APX), facilitating ROS detoxification and enhancing drought tolerance.^[^ ^126^ ^]^ Additionally, the peptide hormones OsRALF45/46 interact with the receptor‐like kinase OsMRLK63, contributing to improved drought resistance.^[^ ^127^ ^]^ The overexpression of the rice gene *OsS1Fa1* enhances drought tolerance in *Arabidopsis*.^[^ ^87^ ^]^ TaCEP1D peptide hormone, a member of the CEP family, enhances drought resistance by binding to CEPR1 and CEPR2 receptors in wheat.^[^ ^128^ ^]^ CEP peptide hormones also play key roles in strengthening drought tolerance in tomato.^[^ ^129^ ^]^ CEP5‐mediated signaling is relevant for osmotic and drought stress tolerance in *Arabidopsis*, with CEP5 counteracting auxin effects.^[^ ^130^ ^]^

Drought stress also triggers maturation of the prePSK peptide precursor through the action of Subtilisin‐like Protease 3.8 (SBT3.8), leading to release of bioactive PSK. Upon binding to the plasma membrane‐localized receptor kinase PSKR, PSK enhances drought‐stress tolerance in *Arabidopsis*.^[^ ^131^ ^]^ Drought‐induced flower drop in tomato was also regulated by PSK. PSK formation in response to drought stress depends on phytaspase 2, a subtilisin‐like protease of the phytaspase subtype that generates the peptide by aspartate‐specific processing of the prePSK precursor in the tomato flower pedicel.^[^ ^132^ ^]^ The mature peptide hormone acts in the abscission zone where it induces expression of cell wall hydrolases that execute the abscission process.^[^ ^132^ ^]^ Plants coordinately respond to drought stress through multiple peptide hormone signaling systems (e.g., CLE25‐BAM1/3, OsDSSR1‐ROS scavenging system, CEP‐CEPR1/2, and PSK‐PSKR), which regulate key physiological processes including ABA biosynthesis, stomatal closure, ROS scavenging, and organ abscission. These findings reveal the multidimensional regulatory roles of peptide hormones in plant drought adaptation. Future research could integrate these peptide‐receptor modules to develop smart drought‐resistant crops based on peptide hormone molecular design, thereby providing innovative solutions to address food security challenges under climate change.

### 6.6 Temperature Tolerance

Peptide hormones play important roles in plant growth under temperature stress.^[^ ^133^ ^]^ In response to cold stress, plants produce miPEPs, which are derived from pri‐miRNAs.^[^ ^134^ ^]^ Specifically, miPEP172b and miPEP3635b enhance cold tolerance by regulating their corresponding miRNA genes.^[^ ^135^ ^]^ Additionally, the CLE45 peptide hormone, which originates from the stigma and travels through the pollen tube, is involved in heat stress acclimation (Figure 4F). The CLE45‐SKM1/SKM2 signaling pathway facilitates pollen performance at elevated temperatures, thereby supporting successful seed production.^[^ ^136^ ^]^

### 6.7 pH Sensing and Regulation

Dynamic changes in pH play a critical role in regulating various aspects of plant growth and development, and emerging evidence suggests that multiple plant peptides are involved in pH‐responsive signaling pathways. The plant signaling peptide Rapid Alkalization Factor (RALF) induces rapid alkalinization of the extracellular matrix in plant cells.^[^ ^137^ ^]^ In *Arabidopsis thaliana*, peptide receptors in the Root Apical Meristem (RAM) can perceive extracellular pH changes. Immune response‐induced extracellular alkalinization enhances the alkaline‐dependent Pep1‐PEPR interaction to promote immune responses.^[^ ^138^ ^]^ In Arabidopsis root meristems, Protophloem Sieve Element (PPSE) cells establish an extracellular “acidic‐to‐alkaline” pH gradient to specifically inhibit the autocrine signaling of CLV3/ESR‐family peptide CLE45, thereby autonomously regulating their differentiation process.^[^ ^139^ ^]^ AtRALF1 inhibits root elongation under low pH conditions by binding to the FERONIA receptor and activating H⁺‐ATPase, thus modulating extracellular pH.^[^ ^140^ ^]^ These researches shed light on how pH regulates plant peptide‐receptor perception. However, whether the pH‐sensing mechanism of plant peptides is conserved across different species remains to be further investigated.

### 6.8 The Balance Between Stress Tolerance and Growth

Plants must make trade‐offs between growth and allocating energy to stress responses to survive in a constantly changing environment. Plants regulate concentrations of PSY peptide hormones to balance growth and stress responses. Under optimal growth conditions, PSY peptide hormones bind to their receptors PSYR1, PSYR2, and PSYR3 (Plant Peptide Containing Sulfated Tyrosine Receptors), inhibiting the downstream signaling pathway and promoting plant growth. When exposed to stress, cellular damage leads to a decrease of PSY peptide hormones in the extracellular concentration in neighboring cells, which allows unbound PSYR receptors to activate the expression of stress resistance‐related genes, thereby triggering the stress response mechanism (Figure 4G). The types of stress that the PSY peptide hormone and its receptor PSYR respond to include salt, high temperature, drought, and biotic stress.^[^ ^11^ ^]^ The PSY‐PSYR module establishes a dynamic balance mechanism between plant growth and stress responses by sensing extracellular PSY peptide concentration changes: elevated PSY levels promote growth, while stress‐induced PSY reduction activates PSYR‐mediated stress resistance. This discovery reveals a molecular switch governing the plant “growth‐defense” trade‐off.

## 7 The Role of Peptide Hormones in Plant Immunity

**Figure 5:**
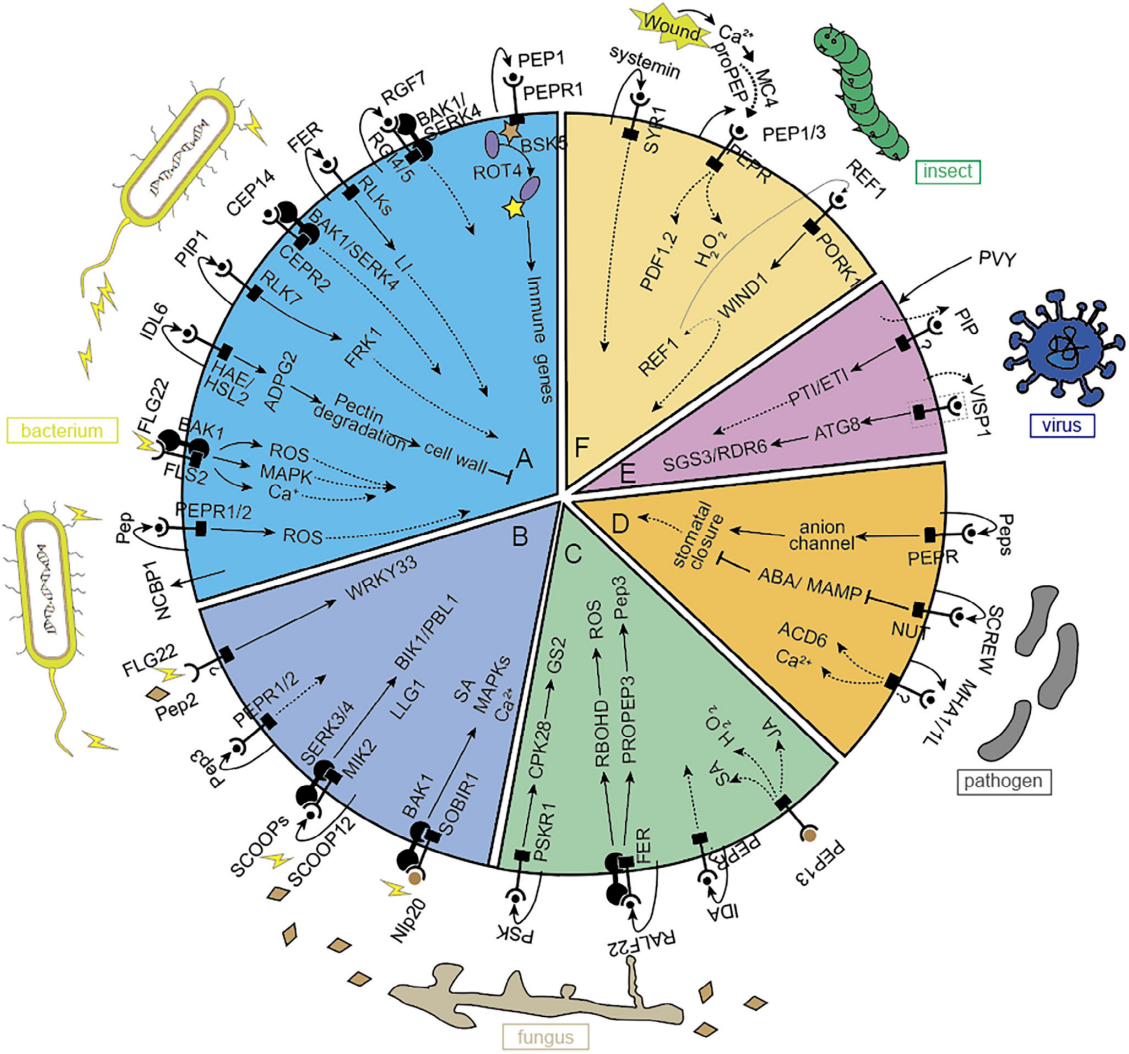
Signaling Pathways of Small Peptides in Plant Defense. Solid arrows represent direct positive regulation within the signaling pathways, which involve in the activation of defense mechanisms or responses triggered by small peptides upon pathogen attack or injury. T‐lines without arrowheads indicate negative regulation, where the presence of small peptides may inhibit certain pathways or responses. Solid lines depict direct interactions or regulatory effects between small peptides, their receptors, and downstream signaling components. Dashed lines indicate pathways involving in multiple steps or components that are not fully elucidated. A) Bacterial immunogen. B) Bacterial and fungal immunogens. C) Fungal immunogen. D) Unclassified pathogen immunogens. E) Virus immunogen. F) Herbivorous insects damage.

Peptide hormones play important roles in regulating plant immune responses.^[^ ^141^ ^]^ The non‐secretory peptide hormone ROT4 interacts with BSK5, disrupting its binding to the receptor kinase PEPR1, which activates the plant immune response dependent on the secretion of PEP peptide hormones. Elevated *ROT4* expression enhances pathogen resistance in *Arabidopsis*.^[^ ^36^ ^]^ The pathogen‐induced peptide hormone RGF7 is perceived by the receptor‐like kinases RGI4 and RGI5, in complex with co‐receptors SERK4 and Brassinosteroid Insensitive 1‐Associated Receptor Kinase 1 (BAK1), initiating innate immunity.^[^ ^142^ ^]^ The aquaporin AtPIP1 associates with RECEPTOR‐LIKE KINASE 7 to induce the transcription of the *FRK1* gene, thereby initiating the plant's immune response.^[^ ^143^ ^]^ Similarly, StPIP1 performs an analogous role in *Solanum tuberosum*.^[^ ^144^ ^]^ PIP1 and PEP1 can synergistically amplify the immune response triggered by the PAMP flagellin.^[^ ^143^ ^]^ A plant‐derived Noncanonical Antibacterial Peptide (NCBP1), composed of 11 amino acid residues with cationic surface potential and favorable safety and stability, is effective against both Gram‐positive and Gram‐negative bacteria. Mechanistic studies revealed that NCBP1 displayed antibacterial activity by targeting phosphatidylglycerol and cardiolipin in the bacterial membrane, resulting in membrane damage and dysfunction^[^ ^145^ ^]^ (**Figure** **5**A).

IDA‐LIKE peptide hormones IDL6 and IDL7 act as negative modulators of stress responses. The IDL6‐HAESA/HAESA‐Like 2 (HAE/HSL2) signaling module stimulates transcription of the polygalacturonase‐encoding gene *ADPG2*, enhances polygalacturonase enzymatic activity, decreases pectin content in leaf cell walls, and reduces the cell wall's defensive function during immune responses.^[^ ^146^ ^]^ Co‐treatment with IDL7 and the bacterial elicitor FLAGELLIN SENSING22 (FLG22) mitigates excessive production of ROS that occurs with FLG22 treatment alone.^[^ ^103^ ^]^ Flagellin‐derived FLG22, a pathogen‐associated molecular pattern (PAMP), is recognized by the plant's cell surface receptor Flagellin Sensing 2 (FLS2) in conjunction with co‐receptor BAK1.^[^ ^147^ ^]^ The binding of FLG22 to FLS2 initiates a cascade of signaling events, including rapid ROS accumulation, inward flux of extracellular calcium ions, and activation of the MPK signaling module, orchestrating the plant's innate immune response^[^ ^148^ ^]^ (Figure 5A). Upon pathogen attack, transcription of the *CLE3* gene is upregulated, leading to activation of the *WRKY33* gene and bolstering the plant's resistance to pathogens^[^ ^149^ ^]^ (Figure 5B). In response to infection by the bacterial pathogen *Pseudomonas syringae*, *CEP14* was highly induced via the salicylic acid (SA) pathway in *Arabidopsis* leaves and roots. SERKs BAK1 and SERK4 participated in CEP14 perception by forming CEP14‐induced complexes with CEPR2. Overexpression of *CEP14* largely enhanced *Arabidopsis* resistance to *Pseudomonas syringae*, while CEP14 or CEPR2 mutation significantly attenuated *Arabidopsis* systemic resistance to *Pseudomonas syringae* ^[^ ^98^ ^]^ (Figure 5A).

NLP20 (Necrosis and Ethylene‐inducing Peptide 1‐like Protein 20) is an important immunogenic peptide in the plant immune system. This class of proteins was initially identified in pathogenic microorganisms (such as fungi and oomycetes) and can induce plant cell death (necrosis) and ethylene synthesis, thereby triggering plant defense responses.^[^ ^150^ ^]^ Arabidopsis LRR receptor protein RLP23 forms a trimeric complex with co‐receptors SOBIR1 and BAK1 to specifically recognize the immunogenic peptide nlp20, which is widely present in bacteria, oomycetes, and fungi, thereby activating plant immune responses.^[^ ^151^ ^]^ Upon perceiving pathogen‐derived elicitors such as flg22 and nlp20, PRRs activate early immune responses including ROS production, MAPK activation, and Ca^2^⁺ influx through RLCKs. Elevated intracellular Ca^2^⁺ levels induce TIR gene expression, which subsequently activates downstream defense pathways via EDS1/PAD4/ADR1s and EDS1/SAG101/NRG1s signaling modules. The activation of TIR signaling further induces the expression of downstream defense genes, leading to enhanced SA biosynthesis and strengthened pathogen resistance^[^ ^152^ ^]^ (Figure 5B).

The receptor kinase MIK2, located on the plant cell surface, recognizes the SCOOP12 protein, which binds to the co‐receptors SERK3 and SERK4 to induce the formation of a heterodimer complex.^[^ ^153^ ^]^ This complex facilitates signal transduction through the cytoplasmic receptor‐like kinases Botrytis‐Induced Kinase 1 (BIK1) and Avrpphb Susceptible 1 (PBS1)‐Like 1 (PBL1), leading to activation of the plant's immune response.^[^ ^141^ ^]^ SCOOPs also mediate the complex formation of *Arabidopsis* MIK2 and co‐receptor BAK1, triggering immune responses. The absence of N‐glycosylation at the specific site in MIK2 markedly reduces its affinity for BAK1, abolishing SCOOP‐triggered immune responses^[^ ^154^ ^]^ (Figure 5B). In *Brassica* species, the peptide hormone RALF22 forms a heterologous complex with the CrRLK1L receptor kinase FER and the co‐receptor LLG1 to activate the NADPH oxidase RBOHD, leading to the production of ROS that enhances plant resistance to *Sclerotinia sclerotiorum*. Additionally, RALF22 can amplify the Pep3‐induced immune signal by significantly increasing the abundance of PROPEP3 transcripts and proteins^[^ ^155^ ^]^ (Figure 5C). Peptide hormone RALF23 specifically binds to the receptor FER‐Like (FER‐LLG2).^[^ ^156^ ^]^

In *Physcomitrella patens*, knockout of *PpRALF2* and *PpRALF3* genes increases resistance to bacterial and fungal phytopathogens, suggesting that these peptide hormones negatively regulate the immune response^[^ ^157^ ^]^ (Figure 5B). In tomato, the PSK peptide hormones interact with their receptor PSK Receptor 1 (PSKR1) to facilitate interaction with the calcium‐dependent protein kinase Calcium‐Dependent Protein Kinase 28 (CPK28), which leads to phosphorylation of key enzymes in the glutamine synthetase/glutamate synthase (GS/GOGAT) cycle, which is important for balancing plant defense responses and growth^[^ ^158^ ^]^ (Figure 5C).

*Sclerotinia sclerotiorum* (*S. sclerotiorum*) is a major pathogen affecting *Brassica napus* (canola), particularly during flowering.^[^ ^159^, ^160^ ^]^ IDA peptide hormones are involved in regulating flower abscission in canola, thus delaying the onset of *S. sclerotiorum* infection^[^ ^161^ ^]^ (Figure 5C). RALF peptide hormones are also important in canola's immune response against fungal pathogens. Suppressing *BnRALF10*, a RALF peptide hormone in canola, significantly reduces resistance to the pathogen. Conversely, exogenous application of BnRALF10 peptide hormones enhances canola's resistance to *S. sclerotiorum*, indicating their role in modulating plant defense mechanisms against this pathogenic fungus^[^ ^162^ ^]^ (Figure 5C).

In *Arabidopsis*, tissue damage triggers an influx of calcium ions into the cytosol, activating the plant cysteine protease metacaspase family member MC4, leading to cleavage of the precursor protein of prePEP1 anchored to the cytoplasmic side of the vacuolar membrane, resulting in the release of PEP1 peptide hormones (Figure 5F). These released Pep1 peptide hormones are then perceived by two leucine‐rich repeat receptor‐like kinases, PEPR1 and PEPR2.^[^ ^163^ ^]^ The Pep1‐PEPR1/2 interaction triggers the activation of defense genes through the ethylene/jasmonic acid (JA) signaling pathways, including plant defensin1.2, and stimulates the synthesis of H_2_O_2_, further activating the plant's defense response^[^ ^164^ ^]^ (Figure 5C). Additionally, StPEP1 induces expression of resistance genes, enhancing resistance to root‐knot nematode infections in *Solanum tuberosum* leaves.^[^ ^165^ ^]^ In *Oryza sativa*, feeding by the brown planthopper (*Nilaparvata lugens*) induces expression of the PEP precursor gene *prePEP* and the receptor gene *PEPR* in the leaf sheath^[^ ^166^ ^]^ (Figure 5F). High expression of the *OsPep3* peptide hormone significantly enhances resistance to the brown planthopper and to pathogens causing rice blast and bacterial leaf blight^[^ ^166^ ^]^ (Figure 5B). Furthermore, expression of *OsPep3* can boost the immune response in *Triticum aestivum*.^[^ ^166^ ^]^ In tomato, the Systemin Receptor 1 (SYR1) binds to the peptide hormone systemin, initiating the plant's defense response against herbivorous insects (Figure 5F). The pathogen *Botrytis cinerea*, which causes gray mold disease, can induce expression of the *systemin* gene in tomato, suggesting that systemin may also play a role in defense against fungal pathogens^[^ ^32^, ^167^ ^]^ (Figure 5C). Upon plant injury, Regeneration Factor 1 (REF1) binds to its receptor, initiating a signaling cascade that leads to the activation of Wound‐Induced Dedifferentiation 1 (WIND1), which triggers tissue repair processes and initiates an organ regeneration program mediated by Plant Organ Regeneration Kinase 1 (PORK1)^[^ ^168^ ^]^ (Figure 5F).

Potato virus Y (PVY) (a species of *Potyvirus*), poses a significant economic threat because of its broad host range and impact on agricultural crops.^[^ ^169^ ^]^ In potato, PVY infection significantly induces expression of *PAMP‐Induced Peptide* (*PIP*) genes. Overexpression of the *StPIP1* gene in transgenic potato plants enhances resistance to PVY infection. In these plants, genes associated with both pattern‐triggered immunity (PTI) and effector‐triggered immunity (ETI) are upregulated in response to the viral infection. PTI is the first line of defense, involving recognition of microbe‐associated molecular patterns (MAMPs). ETI provides a more targeted response by recognizing specific pathogen effectors. The induction of these immune‐related genes indicates that *StPIP1* plays an important role in activating the plant's defense mechanisms against PVY^[^ ^144^ ^]^ (Figure 5E). Additionally, the virus‐induced peptide hormone VISP1 interacts with the autophagy‐related protein ATG8, facilitating the selective autophagic degradation of the SGS3/RDR6 complex to modulate RNA silencing and the antiviral immune response in plants, with mutants of *VISP1* exhibiting altered antiviral responses^[^ ^170^ ^]^ (Figure 5E).

The peptide hormones Modulator of Hyperactive ACD6 1 (MHA1) and MHA1L (MHA1‐Like) interact with Arabidopsis Calcium‐Dependent Protein Kinase 6 (ACD6), affecting its oligomeric state and calcium ion channel activity to regulate the plant's immune response by modulating intracellular calcium signaling, a critical component of the defense network that controls defense‐related genes and pathogen responses. ACD6 plays a central role in interpreting calcium signals and initiating downstream defense responses, underscoring the importance of MHA1 and MHA1L in plant immunity^[^ ^159^ ^]^ (Figure 5D). The Small Phytocytokines Regulating Defense And Water Loss (SCREWs) peptides interact with their receptor, Plant Screw Unresponsive Receptor (NUT), to negatively regulate the ABA signaling pathway and MAMP responses to inhibit stomatal closure and promote stomatal reopening, facilitating the loss of apoplastic water and thus limiting pathogen colonization.^[^ ^171^ ^]^ This mechanism helps balance defense responses with water management, contributing to the plant's overall immune strategy^[^ ^138^ ^]^ (Figure 5D). These studies reveal that plants coordinate innate immune responses through sophisticated peptide hormone signaling networks (e.g., PEP‐PEPR, RALF‐FER, PSK‐PSKR). These peptide‐receptor systems can not only recognize pathogen‐associated molecular patterns (PAMPs) but also integrate damage signals, calcium signaling, and hormone pathways (e.g., SA/JA), thereby establishing a multi‐layered defense mechanism.

## 8 Discussion

Plant peptide hormones, typically defined as polypeptide chains containing fewer than 100 amino acid residues, play important roles in various aspects of plant growth and development. Research on different peptide hormone families in plants remains in its infancy because of technical limitations in separation and identification. Advances in peptidomics and technology have improved the efficiency of peptide separation, with techniques such as nano‐liquid chromatography‐tandem mass spectrometry, capillary electrophoresis, and microchip electrophoresis enhancing the resolution and sensitivity of peptide profiling.^[^ ^172^, ^173^, ^174^ ^]^ The emergence of predictive tools like SignalP 6.0 and ExamPle has facilitated the discovery and analysis of novel peptides by predicting the presence and location of signal peptides, which are essential for directing peptide hormones to their cellular destinations.^[^ ^175^, ^176^, ^177^ ^]^ However, the depth of omics data and the limitations of current prediction methods pose challenges for peptide hormone research including the difficulty in identifying and characterizing peptide hormones because of their low abundance, rapid degradation, and the complexity of the plant proteome. These obstacles must be overcome to gain a comprehensive understanding of the roles of plant peptide hormones in cellular processes and intercellular communication in the future.

Non‐conventional Peptides (NCPs) in plants refer to small peptide hormones that do not conform to traditional gene coding rules. They are typically derived from short open reading frames (sORFs), originate from non‐coding regions such as long non‐coding RNAs (lncRNAs) or untranslated regions (UTRs), or are produced through non‐canonical translation mechanisms such as ribosomal frameshifting or translation reinitiation.^[^ ^178^ ^]^ Although NCPs originate from previously unannotated coding regions, an increasing number of studies have shown that NCPs play important biological roles in plants. For example, POLARIS (PLS) is located within a short, ≈500‐nucleotide, auxin‐inducible transcript and encodes a predicted peptide of 36 amino acid residues. PLS regulates root growth and leaf vascular patterning by maintaining the auxin–cytokinin balance.^[^ ^179^ ^]^ The ROTUNDIFOLIA4 (ROT4) open reading frame encodes a small peptide not previously annotated in the Arabidopsis genome, and ROT4 controls leaf morphology in Arabidopsis.^[^ ^35^ ^]^ OSIPs are involved in oxidative stress tolerance in Arabidopsis.^[^ ^102^ ^]^ These types of NCPs play essential roles in plant growth and development, stress responses, and intercellular communication. Studying NCPs contributes to a redefinition of the complexity of plant genomes and their regulatory networks.

In the regulatory network of plant peptide hormones, various peptides may have functional redundancy, which enhances the robustness of the system, allowing plants to maintain physiological functions even if some peptide hormones are compromised and making it challenging to pinpoint the precise role of specific peptide hormones. Plant peptide hormones exert their regulatory functions through diverse signaling pathways, involving multiple components and cross‐regulation, including direct or indirect modulation of transcription factor activity and regulation of gene expression by influencing chromatin structure. CLE peptide hormones interact with various receptor kinases to control plant meristem maintenance, cellular differentiation, and pattern formation.^[^ ^180^ ^]^ CLE peptide hormones can activate or repress multiple signaling cascades, including those associated with the WUS and CLV pathways, which are important for developmental processes. The interaction of CLE peptide hormones with their receptors can initiate signal transduction events that lead to the stabilization or degradation of key developmental regulators, orchestrating complex developmental programs within the plant.^[^ ^37^, ^38^, ^39^, ^40^, ^41^ ^]^

Peptide hormones within the same family can have diverse functions. PSKs are a class of disulfated pentapeptides known as plant peptide hormones. PSK‐α, ‐γ, ‐δ, and ‐ϵ are four bioactive PSKs that play roles in plant growth, development, and immunity. These PSKs modulate various physiological processes by interacting with their specific receptors, leading to the activation of downstream signaling pathways involved in cellular differentiation, organogenesis, and defense responses.^[^ ^175^ ^]^ Thus, in‐depth functional analysis of known peptide hormones is essential to elucidate their complex roles and interactions within the intricate regulatory networks of plant biology.

Research has increasingly identified peptide hormones that work in conjunction with plant hormones to regulate plant growth and development. CLE peptides interact with signaling pathways of hormones, such as auxins, cytokinins, gibberellins, and abscisic acid, indicating significant overlap in their regulatory functions.^[^ ^181^ ^]^ During plant reproductive development, a complex regulatory network among small peptides is formed. Peptide hormones such as PCP‐Bs and RALFs participate in male–female interactions, modulating reproductive processes.^[^ ^182^ ^]^ These peptide hormones are important for processes like pollen–stigma recognition, pollen tube growth, and fertilization, which are essential for successful plant reproduction.^[^ ^183^ ^]^

Currently, genetic methods are the most common approach for identifying and studying peptide hormone signals and their interacting molecules in plants. However, gene redundancy because of the multi‐gene family nature of peptide precursors and receptor‐like proteins limits this research method.^[^ ^184^ ^]^ Bioinformatics tools and omics technologies, including peptidomics and multi‐omics analysis, offer powerful means for discovering and studying peptide hormones. Hidden markov model (HMM)‐based search programs have identified 1628 CLE peptide genes from 57 plant genomes and performed clustering analysis, providing valuable insights for phylogenetic and functional studies of CLE peptide hormones across species.^[^ ^185^ ^]^ Examining their expression patterns under various conditions is helpful to elucidate the roles of peptide hormones in plant physiology and their responses to environmental changes.

The 3D structure of plant peptide hormones is a key research focus, offering insights into peptide action mechanisms. Techniques such as X‐ray crystallography and nuclear magnetic resonance spectroscopy provide high‐resolution structural information for these peptides.^[^ ^186^, ^187^ ^]^ Additionally, computational methods, such as molecular dynamics simulations and docking studies, enhance our understanding of peptide‐receptor interactions at the molecular level.^[^ ^188^ ^]^ These approaches collectively facilitate a comprehensive investigation into the structure‐function relationships of plant peptide hormones and their roles in cellular signaling and plant adaptation to biotic and abiotic stresses.

Plant Endogenous Peptides (PEPs) are a class of signaling molecules encoded by small gene families that play central roles in plant immune responses and the regulation of growth and development. Recent studies have revealed that PEPs participate in biotic stress responses by activating pattern‐triggered immunity (PTI) or modulating hormone signaling pathways; however, their functions often exhibit antagonism with plant growth and developmental processes. Through their receptors PEPR1 and PEPR2, two receptor‐like kinases, Peps mediate both the induction of plant immune responses and the inhibition of root growth.^[^ ^115^ ^]^ PEP signaling is also closely associated with the production of reactive oxygen species (ROS), which act as important secondary messengers in plant defense responses. However, excessive accumulation of ROS can lead to cytotoxicity and damage actively growing tissues (Z^[^ ^141^ ^]^). This antagonistic relationship reflects the resource trade‐off between defense and growth in plants. Elucidating the spatiotemporal expression patterns of PEPs in specific tissues, along with their receptor interaction networks, will contribute to the development of breeding strategies that achieve coordinated regulation of disease resistance and growth.

Plant peptide hormones provide plants with flexibility and stability to cope with complex environments through functional redundancy and multifunctionality. Functional redundancy is primarily manifested through gene family expansion. For example, the number of Cysteine‐rich Peptides (CRPs) has expanded in several angiosperms; the loss of a single CLE gene can be functionally compensated by other family members to maintain normal development; and multiple members of the RALF peptide family are involved in regulating cell turgor and growth, exhibiting a certain degree of functional complementation.^[^ ^189^, ^190^ ^]^ This redundancy enhances resistance to mutational perturbations and environmental adaptability. At the same time, many peptide hormones possess the ability to integrate multiple environmental and endogenous signals by interacting with different receptors or performing distinct functions in different tissues, thereby coordinating growth and stress responses. Under normal growth conditions, the RALF1–FER–LLG1 complex promotes cell expansion; under salt stress, RALF1 binds to the FERONIA receptor and modulates the ABA signaling pathway, interacting with PYL ABA receptors to enhance the response to stress signals.^[^ ^5^, ^140^ ^]^ Similarly, the IDA (INFLORESCENCE DEFICIENT IN ABSCISSION) peptide not only regulates organ abscission but also controls stomatal closure under water stress, reflecting its role in environmental signal integration.^[^ ^191^ ^]^ The combination of functional redundancy and pleiotropy in plant peptide hormones reflects an evolutionary adaptation to balance robustness and plasticity.

Plant peptide hormones, as crucial signaling molecules mediating intercellular communication, exhibit high conservation across plant species while displaying a degree of structural and functional diversity. Many well‐characterized peptide families (e.g., CLE, CEP, PSK, RALF) were first identified in model plants, yet their homologs have been found in diverse dicots and monocots, underscoring their broad evolutionary conservation.^[^ ^192^ ^]^ For instance, CLAVATA3/ESR‐related (CLE) peptides are recognized by corresponding receptors in Arabidopsis, rice, maize, and wheat to regulate shoot apical meristem maintenance and organ development, with their core conserved amino acid sequences (e.g., the 13‐mer CLE motif) being critical for functional activity.^[^ ^193^ ^]^ However, despite the conservation of core peptide sequences, their precursor processing, post‐translational modifications (e.g., hydroxylation, sulfation), and expression patterns vary markedly across species or even tissues, leading to functional diversification.^[^ ^194^ ^]^ Spatial expression dynamics in target tissues further influence their biological roles. For example, while the PSK (Phytosulfokine) family promotes cell division and expansion in many plants, its expression in Arabidopsis is primarily localized to root tips and meristems, whereas in tomato, it prominently contributes to disease resistance—highlighting conserved functions with species‐specific biological roles.^[^ ^195^, ^196^ ^]^

Post‐translational modifications (PTMs) play a critical role in the functional regulation of plant small peptides, significantly influencing their signaling capacity, receptor‐binding specificity, and mutual integration among different peptide families.^[^ ^27^ ^]^ Before being processed into mature active forms, small peptides often undergo modifications such as sulfation, hydroxylation, and glycosylation.^[^ ^197^ ^]^ These modifications can enhance peptide stability, prolong their lifespan in the extracellular environment, and improve their diffusion capacity between tissues.^[^ ^198^ ^]^ PTMs can also alter the spatial conformation and charge state of peptide molecules, thereby significantly enhancing their binding affinity to specific receptors such as LRR‐RLK kinases. For instance, sulfation is essential for receptor recognition in PSK‐like peptides.^[^ ^76^ ^]^ PTMs precisely regulate the structure, stability, and receptor‐binding capacity of small peptides, enabling their dynamic regulatory roles in plant development, nutrient acquisition, and stress responses. Future research should further explore the synergistic mechanisms between PTMs and other signaling pathways, which may provide novel strategies for crop stress‐resistance breeding.

Further studies reveal that even homologous peptide hormones within the same family may undergo “neofunctionalization” or “subfunctionalization” across species. The CEP family, for instance, primarily regulates root differentiation in Arabidopsis but also mediates systemic signaling in legume‐rhizobial symbiosis, a divergence likely linked to subtle shifts in receptor affinity or ligand expression patterns.^[^ ^195^ ^]^ Additionally, peptide activity often depends on precise chemical modifications (e.g., hydroxyproline or sulfation), which are critical for receptor recognition in CLE and PSK peptides. Variations in the enzymatic machinery for these modifications among plants further underpin species‐specific functionalities.^[^ ^6^, ^7^, ^8^, ^9^, ^10^, ^11^, ^12^, ^13^, ^14^, ^15^, ^16^, ^17^, ^19^, ^20^, ^21^, ^22^, ^23^, ^24^, ^25^, ^26^, ^27^ ^]^ In summary, plant peptide hormones demonstrate striking evolutionary conservation in core sequences and signaling mechanisms, yet their precursor structures, chemical modifications, spatiotemporal expression, and receptor interactions exhibit species‐specific adaptations. This “diversity within a conserved framework” ensures the universality of peptide signaling in development and stress responses while enabling lineage‐specific functional innovations. These insights provide a theoretical foundation for future peptide research and their precision applications in crop improvement.

Small peptides exhibit diverse origins and sequences, yet they often demonstrate functional synergy, signaling crosstalk, and functional overlap, forming a highly integrated regulatory network that enhances plant development and stress resistance. First, different small peptide families achieve functional integration by acting on key receptors or signaling pathways. For example, the CLE (CLAVATA3/ESR‐RELATED) and CEP (C‐terminally Encoded Peptide) families, despite their distinct functions, both regulate root development by activating related receptor kinases (e.g., CLV1, CEPR1), thereby integrating local growth with systemic nutrient status.^[^ ^120^, ^189^ ^]^ Additionally, certain peptides, such as CLE9, modulate stomatal closure, while CLE25 regulates drought resistance via ABA‐mediated stomatal closure.^[^ ^123^, ^124^ ^]^ Second, functional integration among small peptides is also reflected in their complementary and redundant roles. For instance, RALF (Rapid Alkalinization Factor) and PSK (Phytosulfokine) both regulate cell expansion but exhibit distinct expression patterns and regulatory mechanisms in different tissues or environmental conditions. When one peptide's function is impaired, another can compensate via similar pathways.^[^ ^29^, ^196^ ^]^ Furthermore, functional integration relies on spatiotemporal expression and tissue‐specific coordination. For example, the IDA (INFLORESCENCE DEFICIENT IN ABSCISSION) family plays dual roles in organ abscission and stomatal regulation, highlighting its importance in integrating growth and stress signals.^[^ ^191^ ^]^ In summary, the functional integration of plant small peptide families not only enhances the diversity and flexibility of signaling regulation but also provides plants with greater physiological stability and environmental adaptability.

Research has also highlighted the regulatory functions of peptide hormones in agronomic traits across crops such as rice, maize, potato, and tomato, underscoring their potential for crop genetic improvement.^[^ ^199^ ^]^ Molecular design breeding has recently emerged as a trend in crop breeding, and the significant roles of peptide hormones in plants highlight their potential applications in this field. However, the involvement of different peptide hormones in complex regulatory networks presents challenges for their study and application. Thus, further elucidation of peptide hormone functions and the development of novel research techniques is necessary to clarify the molecular mechanisms of peptide hormone signal transduction in plants and reveal their regulatory networks in crop agronomic traits. Effective application of these peptide hormones could lead to the development of crop varieties with enhanced disease resistance and higher yields.

## Conflict of Interest

The authors declare no conflict of interest.

## Supporting information



Supplemental Table 1

## References

[1] Y. Matsubayashi , Annu. Rev. Plant Biol. 2014, 65, 385. 24779997 10.1146/annurev-arplant-050312-120122

[2] H. Zhang , Z. Han , W. Song , J. Chai , Mol. Plant 2016, 9, 1454. 27743937 10.1016/j.molp.2016.10.002

[3] C. Ryan , G. Pearce , Annu. Rev. Cell Dev. Biol. 1998, 14, 1. 9891776 10.1146/annurev.cellbio.14.1.1

[4] S. Wang , L. Tian , H. Liu , X. Li , J. Zhang , X. Chen , X. Jia , Xu Zheng , S. Wu , Y. Chen , J. Yan , L. Wu , Mol. Plant. 2020, 13, 1078. 32445888 10.1016/j.molp.2020.05.012

[5] L. Wang , T. Yang , B. Wang , Q. Lin , S. Zhu , C. Li , Y. Ma , J. Tang , J. Xing , X. Li , H. Liao , D. Staiger , Z. Hu , F. Yu , Sci. Adv. 2020, 6, aaz1622. 10.1126/sciadv.aaz1622 PMC7314565 32671204

[6] J. Chekan , L. Mydy , M. Pasquale , R. Kersten , Nat. Prod. Rep. 2024, 17, 1020. 10.1039/d3np00042g PMC11253845 38411572

[7] Y. Fang , J. Chang , T. Shi , W. Luo , Y. Ou , D. Wan , J. Li , Int. J. Mol. Sci. 2021, 22, 13372. 34948169 10.3390/ijms222413372 PMC8708909

[8] Y. Yamaguchi , T. Ishida , S. Sawa , J. Exp. Bot. 2016, 67, 4813. 27229733 10.1093/jxb/erw208

[9] G. Wang , M. Fiers , Protoplasma 2010, 240, 33. 20016993 10.1007/s00709-009-0095-y PMC2841256

[10] A. Cheung , Annu. Rev. Plant Biol. 2024, 75, 345. 38424067 10.1146/annurev-arplant-102820-103424 PMC12034098

[11] M. Ogawa‐Ohnishi , T. Yamashita , M. Kakita , T. Nakayama , Y. Ohkubo , Y. Hayashi , Y. Yamashita , T. Nomura , S. Noda , H. Shinohara , Y. Matsubayashi , Science 2022, 378, 175. 36227996 10.1126/science.abq5735

[12] D. Robinson , Y. Ding , L. Jiang , Protoplasma 2016, 253, 31. 26410830 10.1007/s00709-015-0887-1

[13] C. Gruber , Planta Med. 2024, 90, 627. 38843800 10.1055/a-2219-9724 PMC11156498

[14] Y. Hirakawa , S. Sawa , Curr. Opin. Plant Biol. 2019, 51, 81. 31132657 10.1016/j.pbi.2019.04.005

[15] A. Motomitsu , S. Sawa , T. Ishida , Essays Biochem. 2015, 58, 115. 26374891 10.1042/bse0580115

[16] Z. Zhang , H. Han , J. Zhao , Z. Liu , L. Deng , L. Wu , J. Niu , Y. Guo , G. Wang , X. Gou , C. Li , C. Li , C.‐M. Liu , Mol. Hortic. 2025, 5, 7. 39849641 10.1186/s43897-024-00134-y PMC11756074

[17] J. Ali , M. Mukarram , J. Ojo , N. Dawam , R. Riyazuddin , H. A. Ghramh , K. A. Khan , R. Chen , D. Kurjak , A. Bayram , Physiol. Plantarum. 2024, 176, 14307. 10.1111/ppl.14307 38705723

[18] S. Lu , F. Xiao , Int. J. Mol. Sci. 2024, 25, 7627. 39062870 10.3390/ijms25147627 PMC11276966

[19] L. Lu , X. Chen , J. Chen , Z. Zhang , Z. Zhang , Y. Sun , Y. Wang , S. Xie , Y. Ma , Y. Song , R. Zeng , Plant, Cell Environ 2024, 47, 1452. 38233741 10.1111/pce.14819

[20] H. Xie , W. Zhao , W. Li , Y. Zhang , J. Hajný , H. Han , Planta 2022, 255, 72. 35218440 10.1007/s00425-022-03859-6

[21] Z. Pei , L. Zhu , S. Nair , Nat. Commun. 2023, 14, 7734. 38007494 10.1038/s41467-023-43604-5 PMC10676384

[22] H. Hõrak , Plant Cell 2022, 34, 1159. 35234915 10.1093/plcell/koac012 PMC8972282

[23] Y. Hsiao , M. Yamada , Genes (Basel) 2020, 12, 22. 33375648 10.3390/genes12010022 PMC7823343

[24] W. Sin , H. Lam , S. Ngai , Int. J. Mol. Sci. 2022, 23, 8641. 35955768 10.3390/ijms23158641 PMC9369194

[25] M. Ogawa‐Ohnishi , W. Matsushita , Y. Matsubayashi , Nat. Chem. Biol. 2013, 9, 726. 24036508 10.1038/nchembio.1351

[26] K. A. T. Silverstein , W. A. Moskal , H. C. Wu , B. A. Underwood , M. A. Graham , C. D. Town , K. A. VandenBosch , Plant J. 2007, 1, 262. 10.1111/j.1365-313X.2007.03136.x 17565583

[27] Y. Matsubayashi , Genes Cells 2011, 17, 1. 10.1111/j.1365-2443.2011.01569.x 22212512

[28] M. Onrubia , J. Pollier , R. Vanden Bossche , M. Goethals , K. Gevaert , E. Moyano , H. Vidal‐Limon , R. M. Cusidó , J. Palazón , A. Goossens , Plant Biotechnol. J. 2014, 12, 971. 24852175 10.1111/pbi.12205

[29] M. Stegmann , J. Monaghan , E. Smakowska‐Luzan , H. Rovenich , A. Lehner , N. Holton , Y. Belkhadir , C. Zipfel , Science 2017, 355, 287. 28104890 10.1126/science.aal2541

[30] W. Tang , I. Ezcurra , J. Muschietti , S. McCormick , Plant Cell 2002, 14, 2277. 12215520 10.1105/tpc.003103 PMC150770

[31] S. Tian , X. Wang , P. Li , H. Wang , H. Ji , J. Xie , Q. Qiu , D. Shen , H. Dong , Plant Physiol. 2016, 171, 1635. 26945050 10.1104/pp.15.01237 PMC4936539

[32] L. Wang , E. Einig , M. Almeida‐Trapp , M. Albert , J. Fliegmann , A. Mithöfer , H. Kalbacher , G. Felix , Nat. Plants 2018, 4, 152. 29459726 10.1038/s41477-018-0106-0

[33] P. M. Chilley , S. A. Casson , P. Tarkowski , N. Hawkins , K. L.‐C. Wang , P. J. Hussey , M. Beale , J. R. Ecker , G. K. Sandberg , K. Lindsey , Plant Cell 2006, 18, 3058. 17138700 10.1105/tpc.106.040790 PMC1693943

[34] P. Ganguly , D. Roy , T. Das , A. Kundu , F. Cartieaux , Z. Ghosh , M. DasGupta , Mol. Plant Microbe Interact. 2021, 34, 1057. 33934615 10.1094/MPMI-12-20-0357-R

[35] N. N. Narita , S. Moore , G. Horiguchi , M. Kubo , T. Demura , H. Fukuda , J. Goodrich , H. Tsukaya , Plant J. 2004, 38, 699. 15125775 10.1111/j.1365-313X.2004.02078.x

[36] W. Li , T. Ye , W. Ye , J. Liang , W. Wang , D. Han , X. Liu , L. Huang , Y. Ouyang , J. Liao , T. Chen , C. Yang , J. Lai , EMBO Rep. 2024, 25, 489. 38177916 10.1038/s44319-023-00029-x PMC10897394

[37] C. Hu , Y. Zhu , Y. Cui , K. Cheng , W. Liang , Z. Wei , M. Zhu , H. Yin , L.i Zeng , Y.a Xiao , M. Lv , J. Yi , S. Hou , K. He , J. Li , X. Gou , Nat. Plants 2018, 4, 205. 29581511 10.1038/s41477-018-0123-z

[38] R. Müller , A. Bleckmann , Rü Simon , Plant Cell 2008, 20, 934. 18381924 10.1105/tpc.107.057547 PMC2390746

[39] M. Ogawa , H. Shinohara , Y. Sakagami , Y. Matsubayashi , Science 2008, 319, 294. 18202283 10.1126/science.1150083

[40] H. Shinohara , Y. Matsubayashi , Plant J. 2015, 82, 328. 25754504 10.1111/tpj.12817

[41] Y. Zhou , X. Liu , E. M. Engstrom , Z. L. Nimchuk , J. L. Pruneda‐Paz , P. T. Tarr , An Yan , S. A. Kay , E. M. Meyerowitz , Nature 2015, 517, 377. 25363783 10.1038/nature13853 PMC4297503

[42] T. Suzaki , M. Ohneda , T. Toriba , A. Yoshida , H. Hirano , PLoS Genet. 2009, 5, 1000693. 10.1371/journal.pgen.1000693 PMC2752996 19834537

[43] C.‐T. Kwon , L. Tang , X. Wang , I. Gentile , A. Hendelman , G. Robitaille , J. Van Eck , C. Xu , Z. B. Lippman , Nat. Plants 2022, 8, 346. 35347264 10.1038/s41477-022-01118-w

[44] D. Rodriguez‐Leal , C. Xu , C.‐T. Kwon , C. Soyars , E. Demesa‐Arevalo , J. Man , L. Liu , Z. H. Lemmon , D. S. Jones , J. Van Eck , D. P. Jackson , M. E. Bartlett , Z. L. Nimchuk , Z. B. Lippman , Nat. Genet. 2019, 51, 786. 30988512 10.1038/s41588-019-0389-8 PMC7274162

[45] N. Kawamoto , D. P. Del Carpio , A. Hofmann , Y. Mizuta , D. Kurihara , T. Higashiyama , N. Uchida , K. U. Torii , L. Colombo , G. Groth , R. Simon , Curr. Biol. 2020, 30, 4352. 32916111 10.1016/j.cub.2020.08.050

[46] N. M. Doll , S. Royek , S. Fujita , S. Okuda , S. Chamot , A. Stintzi , T. Widiez , M. Hothorn , A. Schaller , N. Geldner , G. Ingram , Science 2020, 367, 431. 31974252 10.1126/science.aaz4131

[47] Y. Yu , W. Li , Y. Liu , Y. Liu , Q. Zhang , Y. Ouyang , W. Ding , Yu Xue , Y. Zou , J. Yan , A. Jia , J. Yan , X. Hao , Y. Gou , Z. Zhai , L. Liu , Y. Zheng , B. Zhang , J. Xu , N. Yang , Y. Xiao , L. Zhuo , Z. Lai , P. Yin , H.‐J. Liu , A. R. Fernie , D. Jackson , J. Yan , Cell 2025, 188, 44. 39536747 10.1016/j.cell.2024.10.030

[48] B. Wybouw , X. Zhang , A. Mähönen , New Phytol. 2024, 243, 851. 38890801 10.1111/nph.19897

[49] W. Chang , H. Chen , G. Jiao , Yi Dou , L. Liu , C. Qu , J. Li , K. Lu , Biomolecules 2022, 12, 1772. 36551200 10.3390/biom12121772 PMC9775962

[50] J. Etchells , S. Turner , Development 2010, 137, 767. 20147378 10.1242/dev.044941

[51] H. Chu , W. Liang , J. Li , F. Hong , Y. Wu , L. Wang , J. Wang , P. Wu , C. Liu , Q. Zhang , J. Xu , D. Zhang , J. Exp. Bot. 2013, 64, 5359. 24043854 10.1093/jxb/ert301

[52] P. Qian , W. Song , M. Zaizen‐Iida , S. Kume , G. Wang , Y.e Zhang , K. Kinoshita‐Tsujimura , J. Chai , T. Kakimoto , Nat. Plants 2022, 8, 817. 35817820 10.1038/s41477-022-01176-0

[53] P. Qian , W. Song , T. Yokoo , A. Minobe , G. Wang , T. Ishida , S. Sawa , J. Chai , T. Kakimoto , Nat. Plants 2018, 4, 1071. 30518839 10.1038/s41477-018-0317-4

[54] J. S. Lee , T. Kuroha , M. Hnilova , D. Khatayevich , M. M. Kanaoka , J. M. McAbee , M. Sarikaya , C. Tamerler , K. U. Torii , Genes Dev. 2012, 26, 126. 22241782 10.1101/gad.179895.111 PMC3273837

[55] J. S. Lee , M. Hnilova , M. Maes , Y.a‐C. L. Lin , A. Putarjunan , S.‐K.i Han , J. Avila , K. U. Torii , Nature 2015, 522, 439. 26083750 10.1038/nature14561 PMC4532310

[56] J. Lu , J. He , X. Zhou , J. Zhong , J. Li , Y. Liang , J. Plant Physiol. 2019, 234, 18. 30660943 10.1016/j.jplph.2019.01.010

[57] F. Zhu , J. Deng , H. Chen , P. Liu , L. Zheng , Q. Ye , R. Li , M. Brault , J. Wen , F. Frugier , J. Dong , T. Wang , Plant Cell 2020, 32, 2855. 32887805 10.1105/tpc.20.00248 PMC7474297

[58] S. Zhu , J. M. Estévez , H. Liao , Y. Zhu , T. Yang , C. Li , Y. Wang , L. Li , X. Liu , J. M. Pacheco , H. Guo , F. Yu , Mol. Plant 2020, 13, 698. 31904511 10.1016/j.molp.2019.12.014

[59] N. Hayashi , T. Tetsumura , S. Sawa , T. Wada , R. Tominaga‐Wada , Plant Biotechnol. 2018, 35, 17. 10.5511/plantbiotechnology.18.0122a PMC6543736 31275033

[60] N. Hayashi , N. Rongkavilit , T. Tetsumura , S. Sawa , T. Wada , R. Tominaga‐Wada , Plant Biotechnol. 2019, 36, 205. 10.5511/plantbiotechnology.19.0725a PMC6854340 31768124

[61] S. Schoenaers , H. K. Lee , M. Gonneau , E. Faucher , T. Levasseur , E. Akary , N. Claeijs , S. Moussu , C. Broyart , D. Balcerowicz , H. AbdElgawad , A. Bassi , D. S. C. Damineli , A. Costa , J. A. Feijó , C. Moreau , E. Bonnin , B. Cathala , J. Santiago , H. Höfte , K. Vissenberg , Nat. Plants 2024, 10, 494. 38467800 10.1038/s41477-024-01637-8 PMC11494403

[62] N. Busatto , U. Salvagnin , F. Resentini , S. Quaresimin , L. Navazio , O. Marin , M. Pellegrini , F. Costa , D. F. Mierke , L. Trainotti , Front. Plant Sci. 2017, 8, 1711. 29075273 10.3389/fpls.2017.01711 PMC5641559

[63] Q. Zhu , Y. Shao , S. Ge , M. Zhang , T. Zhang , X. Hu , Y. Liu , J. Walker , S. Zhang , J. Xu , Nat. Plants 2019, 5, 414. 30936437 10.1038/s41477-019-0396-x

[64] J. Wang , L. Xi , X. N. Wu , S. König , L. Rohr , T. Neumann , J. Weber , K. Harter , W. X. Schulze , Mol. Plant 2022, 15, 1615. 36131543 10.1016/j.molp.2022.09.016

[65] Z. Ge , T. Dresselhaus , L. Qu , Trends Plant Sci. 2019, 24, 978. 31607472 10.1016/j.tplants.2019.09.002

[66] S. Yang , J. Bai , J. Wang , Planta 2020, 251, 109. 32472155 10.1007/s00425-020-03406-1

[67] J. Yue , H. Yang , S. Yang , J. Wang , Tree Physiol. 2020, 40, 1534. 32598454 10.1093/treephys/tpaa077

[68] S. Nakagami , T. Aoyama , Y. Sato , T. Kajiwara , T. Ishida , S. Sawa , Plant J. 2023, 113, 1176. 36628476 10.1111/tpj.16103

[69] C. Delay , N. Imin , M. Djordjevic , J. Exp. Bot. 2013, 64, 5383. 24179096 10.1093/jxb/ert332

[70] Y. Zhu , C. Hu , Y. Cui , Li Zeng , S. Li , M. Zhu , F. Meng , S. Huang , Li Long , J. Yi , J. Li , X. Gou , Mol. Plant 2021, 14, 1119. 33823234 10.1016/j.molp.2021.04.001

[71] Y. Stahl , S. Grabowski , A. Bleckmann , R. Kühnemuth , S. Weidtkamp‐Peters , K. G. Pinto , G. K. Kirschner , J B. Schmid , R. H. Wink , A. Hülsewede , S. Felekyan , C. A. M. Seidel , R. Simon , Curr. Biol. 2013, 23, 362. 23394827 10.1016/j.cub.2013.01.045

[72] Y. Stahl , R. Wink , G. Ingram , R. Simon , Curr. Biol. 2009, 19, 909. 19398337 10.1016/j.cub.2009.03.060

[73] B. Berckmans , G. Kirschner , N. Gerlitz , R. Stadler , R. Simon , Plant Physiol. 2020, 182, 1776. 31806736 10.1104/pp.19.00914 PMC7140941

[74] Yo Matsuzaki , M. Ogawa‐Ohnishi , A. Mori , Y. Matsubayashi , Science 2010, 329, 1065. 20798316 10.1126/science.1191132

[75] W. Song , L.i Liu , J. Wang , Z. Wu , H. Zhang , J. Tang , G. Lin , Y. Wang , X. Wen , W. Li , Z. Han , H. Guo , J. Chai , Cell Res. 2016, 26, 674. 27229311 10.1038/cr.2016.62 PMC4897187

[76] J. Wang , H. Li , Z. Han , H. Zhang , T. Wang , G. Lin , J. Chang , W. Yang , J. Chai , Nature 2015, 525, 265. 26308901 10.1038/nature14858

[77] L. Li , H. Chen , S. Alotaibi , A. Pěnčík , M. Adamowski , O. Novák , J. Friml , Proc. Natl. Acad. Sci. USA 2022, 119, 2121058119. 10.1073/pnas.2121058119 PMC9351349 35878023

[78] S. Velasquez , J. Kleine‐Vehn , Nat. Plants 2016, 2, 16118. 28221376 10.1038/nplants.2016.118

[79] B. Zhang , B. Xin , X. Sun , D. Chao , H. Zheng , L. Peng , X. Chen , L. Zhang , J. Yu , D. Ma , J. Xia , Plant Cell 2024, 36, 383. 37847118 10.1093/plcell/koad269 PMC10827571

[80] Z. Luo , J. Wang , F. Li , Y. Lu , Z. Fang , M. Fu , K. S. Mysore , J. Wen , J. Gong , J. D. Murray , F. Xie , Plant Cell 2023, 35, 776. 36440970 10.1093/plcell/koac340 PMC9940871

[81] Z. Luo , C. Moreau , J. Wang , F. Frugier , F. Xie , New Phytol. 2022, 234, 1547. 35243632 10.1111/nph.18062

[82] S. Roy , I. Torres‐Jerez , S. Zhang , W. Liu , K. Schiessl , D. Jain , C. Boschiero , H.‐K. Lee , N. Krom , P. X. Zhao , J. D. Murray , G. E. D. Oldroyd , W.‐R. Scheible , M. Udvardi , Plant J. 2024, 118, 607. 38361340 10.1111/tpj.16626

[83] M. J. García , M. Angulo , F. J. Romera , C. Lucena , R. Pérez‐Vicente , Front. Plant Sci. 2022, 13, 971773. 36105702 10.3389/fpls.2022.971773 PMC9465050

[84] M. Ito , Y. Tajima , M. Ogawa‐Ohnishi , H. Nishida , S. Nosaki , M. Noda , N. Sotta , K. Kawade , T. Kamiya , T. Fujiwara , Y. Matsubayashi , T. Suzaki , Nat. Commun. 2024, 15, 733. 38286991 10.1038/s41467-024-44865-4 PMC10825120

[85] C. Lim , Y. Lee , C. Hwang , Plant Cell Physiol. 2011, 52, 1613. 21757457 10.1093/pcp/pcr091

[86] B. Ferguson , D. Li , A. Hastwell , D. Reid , Y. Li , S. Jackson , P. Gresshoff , Plant Biotechnol. J. 2014, 12, 1085. 25040127 10.1111/pbi.12216

[87] S. Kim , K. Lee , J. Kwak , D. Kwon , J. Song , H. Seo , Plants 2021, 10, 2181. 34685986 10.3390/plants10102181 PMC8541125

[88] Z. Lan , Z. Song , Z. Wang , L. Li , Y. Liu , S. Zhi , R. Wang , J. Wang , Q. Li , A. Bleckmann , L.i Zhang , T. Dresselhaus , J. Dong , H. Gu , S. Zhong , L.i‐J. Qu , Cell 2023, 186, 4773. 37806310 10.1016/j.cell.2023.09.003 PMC10615786

[89] S. Zhong , L. Li , Z. Wang , Z. Ge , Q. Li , A. Bleckmann , J. Wang , Z. Song , Y. Shi , T. Liu , L. Li , H. Zhou , Y. Wang , Li Zhang , H.‐M. Wu , L. Lai , H. Gu , J. Dong , A. Y. Cheung , T. Dresselhaus , L.‐J. Qu , Science 2022, 375, 290. 35050671 10.1126/science.abl4683 PMC9040003

[90] S. Okuda , H. Tsutsui , K. Shiina , S. Sprunck , H. Takeuchi , R. Yui , R. D. Kasahara , Y. Hamamura , A. Mizukami , D. Susaki , N. Kawano , T. Sakakibara , S. Namiki , K. Itoh , K. Otsuka , M. Matsuzaki , H. Nozaki , T. Kuroiwa , A. Nakano , M. M. Kanaoka , T. Dresselhaus , N. Sasaki , T. Higashiyama , Nature 2009, 458, 357. 19295610 10.1038/nature07882

[91] H. Takeuchi , T. Higashiyama , Nature 2016, 531, 245. 26961657 10.1038/nature17413

[92] X. Zhang , W. Liu , T. T. Nagae , H. Takeuchi , H. Zhang , Z. Han , T. Higashiyama , J. Chai , Nat. Commun. 2017, 8, 1331. 29109411 10.1038/s41467-017-01323-8 PMC5673899

[93] S. Zhong , M. Liu , Z. Wang , Q. Huang , S. Hou , Y.‐C. Xu , Z. Ge , Z. Song , J. Huang , X. Qiu , Y. Shi , J. Xiao , P. Liu , Ya‐L Guo , J. Dong , T. Dresselhaus , H. Gu , Li‐J Qu , Science 2019, 364, aau9564. 10.1126/science.aau9564 PMC7184628 31147494

[94] L‐Zi Zhou , L. Wang , X. Chen , Z. Ge , J. Mergner , X. Li , B. Küster , G. Längst , Li‐J Qu , T. Dresselhaus , Plant Cell 2024, 36, 1673. 38142229 10.1093/plcell/koad324 PMC11062432

[95] Q. Gao , C. Wang , Y. Xi , Q. Shao , C. Hou , L. Li , S. Luan , Cell Res. 2023, 33, 71. 36588121 10.1038/s41422-022-00754-3 PMC9810639

[96] M. Márton , A. Fastner , S. Uebler , T. Dresselhaus , Curr. Biol. 2012, 22, 1194. 22633810 10.1016/j.cub.2012.04.061

[97] S. Zheng , F. Wang , Z. Liu , H. Zhang , L. Zhang , D. Chen , Genes 2024, 15, 1367. 39596567 10.3390/genes15111367 PMC11593715

[98] X. Wang , W. Yu , Q. Yuan , X. Chen , Y. He , J. Zhou , Q. Xun , G. Wang , J. Li , X. Meng , Plant Physiol. 2024, 197, kiae549. 39412292 10.1093/plphys/kiae549

[99] Z. Ge , T. Bergonci , Y. Zhao , Y. Zou , S. Du , M.‐C. Liu , X. Luo , H. Ruan , L. E. García‐Valencia , S. Zhong , S. Hou , Q. Huang , L. Lai , D. S. Moura , H. Gu , J. Dong , H.‐M. Wu , T. Dresselhaus , J. Xiao , A. Y. Cheung , L.i‐J. Qu , Science 2017, 358, 1596. 29242234 10.1126/science.aao3642 PMC5964610

[100] X. Yu , X. Zhang , P. Zhao , X. Peng , H. Chen , A. Bleckmann , A. Bazhenova , Ce Shi , T. Dresselhaus , M.‐X. Sun , Nature 2021, 592, 433. 33790463 10.1038/s41586-021-03387-5

[101] D. Cabrillac , J. Cock , C. Dumas , T. Gaude , Nature 2001, 410, 220. 11242083 10.1038/35065626

[102] B. De Coninck , D. Carron , P. Tavormina , L. Willem , D. J. Craik , C. Vos , K. Thevissen , J. Mathys , B. P. A. Cammue , J. Exp. Bot. 2013, 64, 5297. 24043855 10.1093/jxb/ert295

[103] A. K. Vie , J. Najafi , P. Winge , E. Cattan , M. Wrzaczek , J. Kangasjärvi , G. Miller , T. Brembu , A. M. Bones , J. Exp. Bot. 2017, 68, 3557. 28586470 10.1093/jxb/erx168 PMC5853212

[104] S. Clemens , J. Plant Physiol. 2006, 163, 319. 16384624 10.1016/j.jplph.2005.11.010

[105] X. Meng , W. Li , R. Shen , P. Lan , J. Hazard. Mater. 2022, 422, 126913. 34419841 10.1016/j.jhazmat.2021.126913

[106] F. Yin , Y. Hu , X. Cao , X. Xiao , M. Zhang , Y. Xiang , L. Wang , Y. Yao , M. Sui , W. Shi , PLoS One 2024, 19, 0312798. 10.1371/journal.pone.0312798 PMC11567544 39546552

[107] F. Xiao , H. Zhou , Front. Plant Sci. 2022, 13, 1053699. 36684765 10.3389/fpls.2022.1053699 PMC9854262

[108] C. Zhao , W. Jiang , O. Zayed , X. Liu , K. Tang , W. Nie , Y. Li , S. Xie , Y. Li , T. Long , L. Liu , Y. Zhu , Y. Zhao , J.‐K. Zhu , Natl. Sci. Rev. 2021, 8, nwaa149. 34691553 10.1093/nsr/nwaa149 PMC8288382

[109] S. Zhao , Q. Zhang , M. Liu , H. Zhou , C. Ma , P. Wang , Int. J. Mol. Sci. 2021, 22, 4609. 33924753 10.3390/ijms22094609 PMC8125386

[110] H. Zhou , F. Xiao , Y. Zheng , G. Liu , Y. Zhuang , Z. Wang , Y. Zhang , J. He , C. Fu , H. Lin , Plant Cell 2022, 34, 927. 34865139 10.1093/plcell/koab292 PMC8824610

[111] K. Nakaminami , M. Okamoto , M. Higuchi‐Takeuchi , T. Yoshizumi , Y. Yamaguchi , Y. Fukao , M. Shimizu , C. Ohashi , M. Tanaka , M. Matsui , K. Shinozaki , M. Seki , K. Hanada , Proc. Natl. Acad. Sci. USA 2018, 115, 5810. 29760074 10.1073/pnas.1719491115 PMC5984501

[112] P. Chien , H. Nam , Y. Chen , J. Exp. Bot. 2015, 66, 5301. 26093145 10.1093/jxb/erv263 PMC4526916

[113] C. Zhao , O. Zayed , Z. Yu , W. Jiang , P. Zhu , C.‐C. Hsu , L. Zhang , W. A Tao , R. Lozano‐Durán , J.‐K. Zhu , Proc. Natl. Acad. Sci. USA 2018, 115, 13123. 30514814 10.1073/pnas.1816991115 PMC6305001

[114] Y. Yu , S. Assmann , Plant Cell Environ. 2018, 41, 2475. 29907954 10.1111/pce.13370 PMC6150805

[115] W. Jiang , Z. Wang , Y. Li , X. Liu , Y. Ren , C. Li , S. Luo , R. M. Singh , Y. Li , C. Kim , C. Zhao , Plant Cell 2024, 36, 4732. 39197037 10.1093/plcell/koae246 PMC11530776

[116] D. Gutiérrez‐Alanís , L. Yong‐Villalobos , P. Jiménez‐Sandoval , F. Alatorre‐Cobos , A. Oropeza‐Aburto , J. Mora‐Macías , F. Sánchez‐Rodríguez , A. Cruz‐Ramírez , L. Herrera‐Estrella , Dev. Cell 2017, 41, 555. 28586647 10.1016/j.devcel.2017.05.009

[117] A. Barragán‐Rosillo , C. Peralta‐Alvarez , J. Ojeda‐Rivera , R. Arzate‐Mejía , F. Recillas‐Targa , L. Herrera‐Estrella , Proc. Natl. Acad. Sci. USA 2021, 118, 2107558118. 10.1073/pnas.2107558118 PMC8379931 34385324

[118] J. Tang , D. Wu , X. Li , L. Wang , L. Xu , Yi Zhang , F. Xu , H. Liu , Q. Xie , S. Dai , D. Coleman‐Derr , S. Zhu , F. Yu , EMBO J. 2022, 41, 109102. 10.15252/embj.2021109102 PMC8922250 35146778

[119] E. Oh , P. Seo , J. Kim , Trends Plant Sci. 2018, 23, 337. 29366684 10.1016/j.tplants.2017.12.007

[120] R. Tabata , K. Sumida , T. Yoshii , K. Ohyama , H. Shinohara , Y. Matsubayashi , Science 2014, 346, 343. 25324386 10.1126/science.1257800

[121] M. Heino , P. Kinnunen , W. Anderson , D. K. Ray , M. J. Puma , O. Varis , S. Siebert , M. Kummu , Sci. Rep. 2023, 13, 3583. 36869041 10.1038/s41598-023-29378-2 PMC9984494

[122] M. Santini , S. Noce , M. Antonelli , L. Caporaso , Sci. Rep. 2022, 12, 5792. 35388057 10.1038/s41598-022-09611-0 PMC8986840

[123] F. Takahashi , T. Suzuki , Y. Osakabe , S. Betsuyaku , Y. Kondo , N. Dohmae , H. Fukuda , K. Yamaguchi‐Shinozaki , K. Shinozaki , Nature 2018, 556, 235. 29618812 10.1038/s41586-018-0009-2

[124] L. Zhang , X. Shi , Y. Zhang , J. Wang , J. Yang , T. Ishida , W. Jiang , X. Han , J. Kang , X. Wang , L. Pan , S. Lv , B. Cao , Y. Zhang , J. Wu , H. Han , Z. Hu , L. Cui , S. Sawa , J. He , G. Wang , Plant Cell Environ. 2019, 42, 1033. 30378140 10.1111/pce.13475

[125] X. Zheng , S. Kang , Y. Jing , Z. Ren , L. Li , J.‐M. Zhou , G. Berkowitz , J. Shi , A. Fu , W. Lan , F. Zhao , S. Luan , Plant Cell 2018, 30, 1132. 29716993 10.1105/tpc.17.00701 PMC6002199

[126] Y. Cui , M. Li , X. Yin , S. Song , G. Xu , M. Wang , C. Li , C. Peng , X. Xia , Plant Sci. 2018, 270, 85. 29576089 10.1016/j.plantsci.2018.02.015

[127] X.‐Q. Jing , P.‐T. Shi , R. Zhang , M.‐R.u Zhou , A. Shalmani , G.‐F. Wang , W.‐T. Liu , W.‐Q. Li , K.‐M. Chen , Plant Physiol. 2024, 194, 2679. 38146904 10.1093/plphys/kiad684

[128] D. Tian , Qi Xie , Z. Deng , J. Xue , W. Li , Z. Zhang , Y. Dai , Bo Zheng , T. Lu , I. De Smet , Y. Guo , Front. Plant Sci. 2022, 13, 1000297. 36212358 10.3389/fpls.2022.1000297 PMC9532867

[129] K. Xu , D. Tian , T. Wang , A. Zhang , M. A. Y. Elsadek , W. Liu , L. Chen , Y. Guo , Mol. Horticul. 2023, 3, 17. 10.1186/s43897-023-00063-2 PMC10515272 37789434

[130] S. Smith , S. Zhu , L. Joos , I. Roberts , N. Nikonorova , L. D. Vu , E. Stes , H. Cho , A. Larrieu , W. Xuan , B. Goodall , B. van de Cotte , J. M. Waite , A. Rigal , S. Ramans Harborough , G. Persiau , S. Vanneste , G. K. Kirschner , E. Vandermarliere , L. Martens , Y. Stahl , D. Audenaert , J. Friml , G. Felix , R. Simon , M. J. Bennett , A. Bishopp , G. De Jaeger , K. Ljung , S. Kepinski , et al., Mol. Cell Proteomics 2020, 19, 1248. 32404488 10.1074/mcp.RA119.001826 PMC8011570

[131] N. Stührwohldt , E. Bühler , M. Sauter , A. Schaller , J. Exp. Bot. 2021, 72, 3427. 33471900 10.1093/jxb/erab017

[132] S. Reichardt , H. Piepho , A. Stintzi , A. Schaller , Science 2020, 367, 1482. 32217727 10.1126/science.aaz5641

[133] G. Ren , Y. Zhang , Z. Chen , X. Xue , H. Fan , Int. J. Mol. Sci. 2024, 25, 4114. 38612923 10.3390/ijms25074114 PMC11012589

[134] D. Lauressergues , M. Ormancey , B. Guillotin , H. San Clemente , L. Camborde , C. Duboé , S. Tourneur , P. Charpentier , A. Barozet , A. Jauneau , A. Le Ru , P. Thuleau , V. Gervais , S. Plaza , J.‐P. Combier , Cell Rep. 2022, 38, 110339. 35139385 10.1016/j.celrep.2022.110339

[135] Q.‐J.u Chen , L.i‐P. Zhang , S.‐R. Song , L. Wang , W.‐P. Xu , C.‐X.i Zhang , S.‐P. Wang , H.‐F. Liu , C. Ma , Plant Sci. 2022, 325, 111450. 36075277 10.1016/j.plantsci.2022.111450

[136] S. Endo , H. Shinohara , Y. Matsubayashi , H. Fukuda , Curr. Biol. 2013, 23, 1670. 23910659 10.1016/j.cub.2013.06.060

[137] L. Liu , X. Liu , Z. Bai , M. Tanveer , Y. Zhang , W. Chen , S. Shabala , L. Huang , Plant Sci. 2024, 343, 112085. 38588983 10.1016/j.plantsci.2024.112085

[138] L.i Liu , W. Song , S. Huang , K. Jiang , Y. Moriwaki , Y. Wang , Y. Men , D. Zhang , X. Wen , Z. Han , J. Chai , H. Guo , Cell 2022, 185, 3341. 35998629 10.1016/j.cell.2022.07.012

[139] H. N. Diaz‐Ardila , B. Gujas , Q. Wang , B. Moret , C. S. Hardtke , Curr. Biol. 2023, 33, 597. 36693368 10.1016/j.cub.2022.12.056

[140] M. Haruta , G. Sabat , K. Stecker , B. B. Minkoff , M. R. Sussman , Science 2014, 343, 408. 24458638 10.1126/science.1244454 PMC4672726

[141] S. Hou , D. Liu , S. Huang , D. Luo , Z. Liu , Q. Xiang , P. Wang , R. Mu , Z. Han , S. Chen , J. Chai , L. Shan , P. He , Nat. Commun. 2021, 12, 5494. 34535661 10.1038/s41467-021-25580-w PMC8448819

[142] X. Wang , Na Zhang , L. Zhang , Y. He , C. Cai , J. Zhou , J. Li , X. Meng , New Phytol. 2021, 230, 1110. 33454976 10.1111/nph.17197

[143] S. Hou , X. Wang , D. Chen , X. Yang , M. Wang , D. Turrà , A. Di Pietro , W. Zhang , PLoS Pathog. 2014, 10, 1004331. 10.1371/journal.ppat.1004331 PMC4154866 25188390

[144] M. M. Combest , N. Moroz , K. Tanaka , C. J. Rogan , J. C. Anderson , L. Thura , A. M. Rakotondrafara , A. Goyer , J. Exp. Bot. 2021, 72, 4472. 33681961 10.1093/jxb/erab078

[145] X. Chen , M. Song , L. Tian , X. Shan , C. Mao , M. Chen , J. Zhao , A. Sami , H. Yin , U. Ali , J. Shi , H. Li , Y. Zhang , J. Zhang , S. Wang , C.‐L. Shi , Y. Chen , X.‐D. Du , K. Zhu , L. Wu , Sci. Adv. 2025, 12, adt8239. 10.1126/sciadv.adt8239 PMC11922054 40106560

[146] X. Wang , S. Hou , Q. Wu , M. Lin , B. Acharya , D. Wu , W. Zhang , Plant J. 2017, 89, 250. 27618493 10.1111/tpj.13380

[147] D. Chinchilla , C. Zipfel , S. Robatzek , B. Kemmerling , T. Nürnberger , J. D. G. Jones , G. Felix , T. Boller , Nature 2007, 448, 497. 17625569 10.1038/nature05999

[148] Y. Chi , C. Wang , M. Wang , Di Wan , F. Huang , Z. Jiang , B. M. Crawford , T. Vo‐Dinh , F. Yuan , F. Wu , Z.‐M. Pei , Plant, Cell Environ. 2021, 44, 3793. 10.1111/pce.14186 34536020

[149] D. Ma , S. Endo , E. Betsuyaku , T. Fujiwara , S. Betsuyaku , H. Fukuda , Plant Mol. Biol. 2022, 108, 225. 35038066 10.1007/s11103-021-01234-9

[150] S. Oome , T. M. Raaymakers , A. Cabral , S. Samwel , H. Böhm , I. Albert , T. Nürnberger , G. Van Den Ackerveken , G. Van den Ackerveken , Proc. Natl. Acad. Sci. USA 2014, 111, 16955. 25368167 10.1073/pnas.1410031111 PMC4250136

[151] I. Albert , H. Böhm , M. Albert , C. E. Feiler , J. Imkampe , N. Wallmeroth , C. Brancato , T. M. Raaymakers , S. Oome , H. Zhang , E. Krol , C. Grefen , A. A. Gust , J. Chai , R. Hedrich , G. Van den Ackerveken , T. Nürnberger , Nat. Plants. 2015, 1, 15140. 27251392 10.1038/nplants.2015.140

[152] H. Tian , Z. Wu , S. Chen , K. Ao , W. Huang , H. Yaghmaiean , T. Sun , F. Xu , Y. Zhang , S. Wang , X. Li , Y. Zhang , Nature 2021, 598, 500. 34544113 10.1038/s41586-021-03987-1

[153] M.‐C. Guillou , E. Vergne , S. Aligon , S. Pelletier , F. Simonneau , A. Rolland , S. Chabout , G. Mouille , K. Gully , P. Grappin , F. Montrichard , S. Aubourg , J.‐P. Renou , J. Exp. Bot. 2022, 73, 6115. 35639812 10.1093/jxb/erac240

[154] H. Wu , L. Wan , Z. Liu , Y. Jian , C. Zhang , X. Mao , Z. Wang , Q. Wang , Y. Hu , L. Xiong , Z. Xia , J. Xue , S. Li , P. He , L. Shan , S. Xu , Nat. Plants 2024, 10, 1984. 39511418 10.1038/s41477-024-01836-3 PMC11649560

[155] Y. He , S. Chen , X. Chen , Y. Xu , Y. Liang , X. Cai , J. Integr. Plant Biol. 2023, 65, 2519. 37698076 10.1111/jipb.13566

[156] Yu Xiao , M. Stegmann , Z. Han , T. A. Defalco , K. Parys , Li Xu , Y. Belkhadir , C. Zipfel , J. Chai , Nature 2019, 572, 270. 31291642 10.1038/s41586-019-1409-7

[157] A. Mamaeva , I. Lyapina , A. Knyazev , N. Golub , T. Mollaev , E. Chudinova , S. Elansky , V. V. Babenko , V. A. Veselovsky , K. M. Klimina , T. Gribova , D. Kharlampieva , V. Lazarev , I. Fesenko , Front. Plant Sci. 2023, 14, 1077301. 36818838 10.3389/fpls.2023.1077301 PMC9933782

[158] S. Ding , J. Lv , Z. Hu , J. Wang , P. Wang , J. Yu , C. H. Foyer , K. Shi , EMBO J. 2023, 42, 111858. 10.15252/embj.2022111858 PMC10015362 36562188

[159] J. Chen , L. Li , J. H. Kim , B. Neuhäuser , M. Wang , M. Thelen , R. Hilleary , Y. Chi , L. Wei , K. Venkataramani , M. Exposito‐Alonso , C. Liu , J. Keck , A. C. Barragan , R. Schwab , U. Lutz , Z.‐M. Pei , S.‐Y. He , U. Ludewig , D. Weigel , W. Zhu , Mol. Cell 2023, 83, 4386. 37995686 10.1016/j.molcel.2023.10.030

[160] R. Chen , J. Wang , R. Sarwar , X. Tan , Front. Plant Sci. 2023, 14, 1276055. 38078117 10.3389/fpls.2023.1276055 PMC10701745

[161] J. Wu , H. Liu , S. Ren , P. Li , X. Li , Li Lin , Q. Sun , L. Zhang , C. Lin , Y. Wang , Plant Physiol. 2022, 190, 1562. 35951752 10.1093/plphys/kiac364 PMC9614459

[162] Y. He , Z. Zhang , Y. Xu , S. Chen , X. Cai , Front. Plant Sci. 2022, 13, 877404. 35592581 10.3389/fpls.2022.877404 PMC9113046

[163] T. Hander , Á. D. Fernández‐Fernández , R. P. Kumpf , P. Willems , H. Schatowitz , D. Rombaut , A.n Staes , J. Nolf , R. Pottie , P. Yao , A. Gonçalves , B. Pavie , T. Boller , K. Gevaert , F. Van Breusegem , S. Bartels , S. Stael , Science 2019, 363, aar7486. 10.1126/science.aar7486 30898901

[164] A. Huffaker , G. Pearce , C. Ryan , Proc. Natl. Acad. Sci. USA 2006, 103, 10098. 16785434 10.1073/pnas.0603727103 PMC1502512

[165] L. Zhang , C. Gleason , Nat. Plants 2020, 6, 625. 32514146 10.1038/s41477-020-0689-0

[166] W. Shen , X. Zhang , J. Liu , K. Tao , C. Li , S. Xiao , W. Zhang , J.‐F. Li , Plant Biotechnol. J. 2022, 20, 991. 35068048 10.1111/pbi.13781 PMC9055822

[167] J. Pastor‐Fernández , N. Sanmartín , M. Manresa‐Grao , C. Cassan , P. Pétriacq , Y. Gibon , J. Gamir , B. Romero‐Rodriguez , A. G. Castillo , M. Cerezo , V. Flors , P. Sánchez‐Bel , J. Exp. Bot. 2024, 75, 4111. 38581374 10.1093/jxb/erae146

[168] W. Yang , H. Zhai , F. Wu , L. Deng , Yu Chao , X. Meng , Q. Chen , C. Liu , X. Bie , C. Sun , Y. Yu , X. Zhang , X. Zhang , Z. Chang , M. Xue , Y. Zhao , X. Meng , B. Li , X. Zhang , D. Zhang , X. Zhao , C. Gao , J. Li , C. Li , Cell 2024, 187, 3024. 38781969 10.1016/j.cell.2024.04.040

[169] A. Inoue‐Nagata , R. Jordan , J. Kreuze , F. Li , J. López‐Moya , K. Mäkinen , K. Ohshima , S. Wylie , Ictv Report Consortium , J. Gen. Virol. 2022, 103, 10.1099/jgv.0.001738. PMC12642016 35506996

[170] X. Tong , S‐Yu Liu , J‐Ze Zou , J.‐J. Zhao , F.‐F. Zhu , L.‐X. Chai , Y. Wang , C. Han , X.‐B. Wang , EMBO J. 2021, 40, 108050. 10.15252/embj.2021108050 PMC8327956 34155657

[171] Z. Liu , S. Hou , O. Rodrigues , P. Wang , D. Luo , S. Munemasa , J. Lei , J. Liu , F. A. Ortiz‐Morea , X. Wang , K. Nomura , C. Yin , H. Wang , W. Zhang , K. Zhu‐Salzman , S. Y. He , P. He , L. Shan , Nature 2022, 605, 332. 35508659 10.1038/s41586-022-04684-3 PMC9710542

[172] M. Pergande , S. Cologna , Proteomes 2017, 5, 4. 28248255 10.3390/proteomes5010004 PMC5372225

[173] G. Scriba , A. Psurek , Methods Mol. Biol. 2008, 384, 483. 18392581 10.1007/978-1-59745-376-9_19

[174] H. Song , X. Wang , W. Hu , X. Yang , E. Diao , T. Shen , Q. Qiang , Food Chem. 2017, 232, 434. 28490095 10.1016/j.foodchem.2017.04.045

[175] Y. Li , Q. Di , L. Luo , L. Yu , Front. Plant Sci. 2023, 14, 1326964. 38250441 10.3389/fpls.2023.1326964 PMC10796568

[176] F. Teufel , J. J. Almagro Armenteros , A. R. Johansen , M. H. Gíslason , S. I. Pihl , K. D. Tsirigos , O. Winther , S. Brunak , G. Von Heijne , H. Nielsen , Nat. Biotechnol. 2022, 40, 1023. 34980915 10.1038/s41587-021-01156-3 PMC9287161

[177] Z. Li , J. Jin , Y. Wang , W. Long , Y. Ding , H. Hu , L. Wei , Bioinformatics 2023, 39, btad108. 36897030 10.1093/bioinformatics/btad108 PMC10027287

[178] M. S. Pei , H. N. Liu , T. L. Wei , Y. H. Yu , D. L. Guo , Hortic. Res. 2022, 9, uhac023. 35531313 10.1093/hr/uhac023 PMC9070638

[179] S. A. Casson , P. M. Chilley , J. F. Topping , I. M. Evans , M. A. Souter , K. Lindsey , Plant Cell 2002, 14, 1705. 12172017 10.1105/tpc.002618 PMC151460

[180] X. Song , X. Hou , C. Liu , Planta 2021, 255, 5. 34841457 10.1007/s00425-021-03791-1

[181] C. Whitewoods , Semin. Cell Dev. Biol. 2021, 109, 12. 32444290 10.1016/j.semcdb.2020.04.022

[182] S. Zhong , L. Qu , Curr. Opin. Plant Biol. 2019, 51, 7. 30999163 10.1016/j.pbi.2019.03.004

[183] C. Liu , L. Shen , Y.u Xiao , D. Vyshedsky , C. Peng , X. Sun , Z. Liu , L. Cheng , H. Zhang , Z. Han , J. Chai , H.‐M. Wu , A. Y. Cheung , C. Li , Science 2021, 372, 171. 33833120 10.1126/science.abc6107

[184] Y. Amano , H. Tsubouchi , H. Shinohara , M. Ogawa , Y. Matsubayashi , Proc. Natl. Acad. Sci. USA 2007, 104, 18333. 17989228 10.1073/pnas.0706403104 PMC2084343

[185] D. Goad , C. Zhu , E. Kellogg , New Phytol. 2017, 216, 605. 27911469 10.1111/nph.14348

[186] L. S. Mydy , J. Hungerford , D. N. Chigumba , J. R. Konwerski , S. C. Jantzi , D.i Wang , J. L. Smith , R. D. Kersten , Nat. Chem. Biol. 2024, 20, 530. 38355722 10.1038/s41589-024-01552-1 PMC11049724

[187] M. Vincenzi , F. Mercurio , M. Leone , Curr. Med. Chem. 2021, 28, 2729. 32614739 10.2174/0929867327666200702131032

[188] J. Peng , N. Svetec , H. Molina , L. Zhao , Mol. Biol. Evol. 2024, 41, msae065. 38518286 10.1093/molbev/msae065 PMC11017328

[189] J. Jun , E. Fiume , A. H. K. Roeder , L. Meng , V. K. Sharma , K. S. Osmont , C. Baker , C. M. Ha , E. M. Meyerowitz , L. J. Feldman , J. C. Fletcher , Plant Physiol. 2010, 154, 1721. 20884811 10.1104/pp.110.163683 PMC2996011

[190] X. Liu , H. Zhang , H. Jiao , L. Li , X. Qiao , M. R. Fabrice , J. Wu , S. Zhang , BMC Genomics 2017, 18, 610. 28806914 10.1186/s12864-017-3948-3 PMC5557327

[191] O. R. Patharkar , J. C. Walker , Plant Physiol. 2016, 172, 510. 27468996 10.1104/pp.16.01004 PMC5074626

[192] C. Furumizu , R. B. Aalen , New Phytol. 2023, 238, 977. 36811171 10.1111/nph.18827

[193] T. Suzaki , A. Yoshida , H. Y. Hirano , Plant Cell 2008, 20, 2049. 18676878 10.1105/tpc.107.057257 PMC2553609

[194] S. Okuda , Peptides 2021, 144, 170614. 34332962 10.1016/j.peptides.2021.170614

[195] S. Ding , S. Feng , S. Zhou , Z. Zhao , X. Liang , J. Wang , R. Fu , R. Deng , T. Zhang , S. Shao , J. Yu , C. H. Foyer , K. Shi , EMBO J. 2024, 43, 6104. 39448885 10.1038/s44318-024-00278-z PMC11612273

[196] A. Kutschmar , G. Rzewuski , N. Stührwohldt , G. T. S. Beemster , D. Inzé , M. Sauter , New Phytol. 2009, 181, 820. 19076296 10.1111/j.1469-8137.2008.02710.x

[197] R. Tabata , S. Sawa , Front. Plant Sci. 2014, 5, 311. 25071794 10.3389/fpls.2014.00311 PMC4082320

[198] S. Royek , M. Bayer , J. Pfannstiel , J. Pleiss , G. Ingram , A. Stintzi , A. Schaller , Proc. Natl. Acad. Sci. USA 2022, 119, 2201195119. 10.1073/pnas.2201195119 PMC9169856 35412898

[199] Y. Feng , Q. Zhu , J. Xue , P. Chen , Y. Yu , aBIOTECH 2023, 4, 238. 37970469 10.1007/s42994-023-00100-0 PMC10638237

